# Elucidation of Dysregulated Pathways Associated With Hypoxia in Oestrogen Receptor‐Negative Breast Cancer

**DOI:** 10.1002/cam4.70274

**Published:** 2024-12-11

**Authors:** Suad A. K. Shamis, Jean Quinn, Sara Al‐Badran, Molly McKenzie, Phimmada Hatthakarnkul, Gerard Lynch, Guang‐Yu Lian, Warapan Numprasit, Laszlo Romics, Ditte Andersen, Elizabeth Mallon, Donald C. McMillan, Joanne Edwards

**Affiliations:** ^1^ Academic Unit of Surgery, School of Medicine University of Glasgow, Royal Infirmary Glasgow UK; ^2^ Wolfson Wohl Cancer Research Centre, School of Cancer Sciences University of Glasgow Glasgow UK; ^3^ NHS Greater Glasgow and Clyde (New Victoria Hospital) Glasgow UK; ^4^ BioClavis Ltd., Teaching & Learning Centre Queen Elizabeth University Hospital Glasgow UK; ^5^ Department of Pathology Queen Elizabeth University Hospital Glasgow UK

**Keywords:** breast cancer, carbonic anhydrases IX, hypoxia, RNA sequencing, survival

## Abstract

**Purpose:**

Carbonic anhydrase IX (CAIX) is a well‐established prognostic marker in breast cancer (BC). Nevertheless, this prognostic value is yet to be confirmed in BC subtypes. This study aims to investigate the prognostic effects of CAIX in oestrogen receptor (ER)‐negative (ER−) BCs and to establish pathways related to cytoplasmic CAIX expression in ER− and lymph node‐negative BCs.

**Methods:**

Immunohistochemistry was performed to identify the prognostic role of CAIX protein expression in ER− tissue microarrays (TMAs) (*n* = 191). CAIX‐positive samples (*n* = 37) were transcriptionally profiled by TempO‐Seq and analysed by STRING. Real‐time quantitative PCR (RT‐qPCR) analysis was used to validate differentially expressed genes.

**Results:**

Overexpression of cytoplasmic CAIX was an independent predictor of recurrence free survival, disease‐free survival and overall survival in ER− cohort. RNA transcriptomic analysis identified 10 significant genes in ER− cohort and 3 genes in the node‐negative group. The STRING database demonstrated a significant interaction between MUCL1 and GALNT6, which were linked with extracellular matrix organisation, degradation of the extracellular matrix and disease of glycosylation pathways. In the node‐negative group, SPNS2 is mainly involved in the sphingolipid de novo biosynthesis pathway. A significant correlation between cytoplasmic SphK1 and cytoplasmic hypoxia‐inducible factor‐1α was observed. Among the 10 genes, 7 genes (SERHL2, GALNT6, MUCL1, MMP7, PITX2, CEACAM6 and SPNS2) were selected, and their expression was quantitatively assessed by RT‐qPCR. The PCR data of these genes showed that SERHL2, GALNT6, MUCL1, PITX2, and SPNS2 mRNA levels were expressed in MDA‐MB‐231 BC cell lines at variable levels of hypoxic exposure.

**Conclusion:**

Cytoplasmic CAIX was independently associated with poor prognosis in ER− BC. Gene expression profiles shed light on the pathways and genes associated with hypoxia in ER− BC. In node‐negative patients, SPNS2 was of particular interest.

**Abbreviations**
ΔCt: delta cycle threshold
95% CI: 95% confidence interval
BC: breast cancer
CAIX: carbonic anhydrase IX
DEGs: differentially expressed genes
DFS: disease‐free survival
ER: oestrogen receptor
FDR: false discovery rate
FFPE: formalin‐fixed paraffin‐embedded
GAPDH: glyceraldehyde‐3‐phosphate dehydrogenase
GO: Gene Ontology
HIF‐1α: hypoxia‐inducible factor‐1α
HR: hazard ratio
ICCC: interclass correlation coefficient
IHC: immunohistochemistry
OS: overall survival
PCA: principal component analysis
PPI: protein–protein interaction
PR: progesterone receptor
RFS: recurrence free survival
RT‐qPCR: real‐time quantitative PCR
S1P: sphingosine 1‐phosphate receptor
SphK1: sphingosine kinase 1
STRING: search tool for the retrieval of interacting genes
TMAs: tissue microarrays
TME: tumour microenvironment
TNBC: triple‐negative breast cancer

## 1 Introduction

Breast cancer (BC) was the most diagnosed cancer and the leading cause of death among women in 2020 [1]. Advances in surgical techniques, improvements in radiation and systemic therapies and screening, and earlier detection have improved patients' survival [2]. However, chemo/radiotherapy resistance and disease metastases remain challenges for BC patients [2, 3, 4]. BC is highly heterogeneous that is categorised into three major categories based on the presence or absence of oestrogen receptor (ER), progesterone receptor (PR) and human epidermal growth factor‐2 (Her‐2) [5]. Approximately 70% of patients had hormone receptor‐positive BC (ER+, PR+, and Her‐2−). Patients with ER‐negative (ER−) BC make up about 30% of all cases and typically have a worse prognosis than ER+ patients [6]. However, a considerable proportion of ER− patients have favourable outcomes and may benefit from less aggressive treatment. Triple‐negative BC (TNBC: ER−, PR− and Her‐2−) makes up 15% of the total number of BCs and has the poorest outcomes.

The hypoxic microenvironment is an important intrinsic component of solid tumours that can result in a rapid proliferation of cancer cells and is associated with the lack of oxygen and abnormal tumour blood vessels [7, 8]. Hypoxia stimulates the hypoxia‐inducible factor‐1α (HIF‐1α) that transactivates genes associated with angiogenesis, tumour growth, metastasis, metabolic reprogramming, immune evasion and treatment resistance [9]. HIF‐1α is recognised to induce the expression of carbonic anhydrase IX (CAIX), an enzyme that has been attributed a central role in pH regulation and cancer progression [10] and is particularly pronounced in peri‐necrotic tumour areas, high‐grade BCs [11, 12]. A recent systematic review and meta‐analyses studied an association between CAIX with BC patient's survival [13].

Multiple transcriptional and post‐transcriptional mechanisms in gene expression control the adaptation to hypoxia. It is estimated that up to 1.5% of the human genome is transcriptionally responsive to hypoxia [14]. Genes and pathways that have been recognised as hypoxia‐responsive have the potential to be used as prognostic or predictive markers, as well as help in the identification of novel treatment targets [15, 16]. Gene profiles might guide treatment decisions for the prospective use of anti‐hypoxic medications in the future, since greater activity of the HIF‐1α pathway is associated with more profound intratumoural hypoxia in TNBC than in other subtypes [14, 17].

In terms of BC subtypes, we recently reported cytoplasmic CAIX expression to be a prognostic marker in luminal B and TNBC [18, 19]. The purpose of the current study was to elucidate the prognostic significance of CAIX in ER− BC and to obtain a better understanding of the transcriptome and protein pathways related to CAIX in ER− and lymph node‐negative BCs, in order to identify potential therapeutic targets for this aggressive phenotype.

## 2 Materials and Methods

The present study was performed in three steps: (1) immunohistochemistry (IHC) of CAIX in ER− BC cohort, (2) transcriptomic analysis of RNA transcripts in ER− tumour tissues and in the node‐negative group and (3) validation of genes by q‐PCR in MDA‐MB‐231 cells.

### 2.1 Patient Cohorts and Tumour Specimens

**FIGURE 1:**
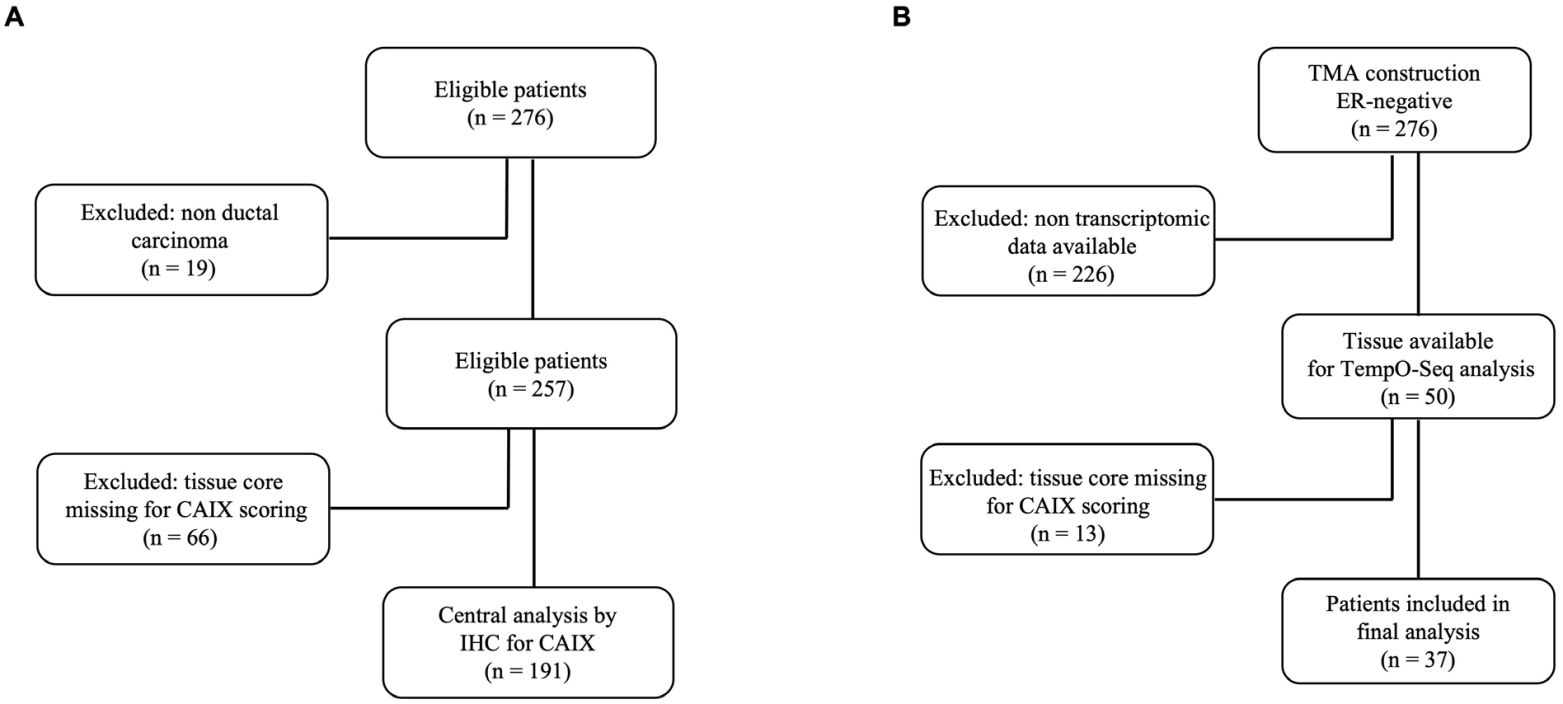
CONSORT diagram of patient inclusion in the study. (A) Selection of 191 ER− BC patients for IHC staining for CAIX and (B) selection of 37 node negative patients for TempO‐Seq analysis.

257 ER− BC patients with ductal carcinoma who had undergone surgical resection at Glasgow Royal Infirmary, Western Infirmary or Stobhill Hospitals (Glasgow, UK) between 1995 and 1998 were included in the study. Of those, 191 patients were selected for IHC to identify the prognostic role of CAIX protein expression in the tumour core of the tissue microarrays (TMAs) (Figure 1A).

Single tissue sections from ER− BC patients were used for templated oligo‐sequencing (TempO‐Seq) analysis using a whole transcriptome panel (*n* = 50). Of these 50 samples, 37 had linked cytoplasmic CAIX protein expression data and were used for final analysis (16 samples had high expression and 21 samples had low expression, Figure 1B). Twenty of these 37 patients had lymph node‐negative and were selected specifically to identify a gene expression signature associated with tumour hypoxia. Patients who received neoadjuvant therapy were excluded.

Patients were routinely followed up after surgery. The date and cause of death were cross‐checked with the cancer registration system and the Registrar General (Scotland). Clinicopathological data were retrieved from the routine reports. This work was approved by The Research Ethics Committee of North Glasgow University Hospitals (NHS GG&C REC reference: 16/WS/0207), and all methods were performed by the relevant guidelines and regulations.

### 2.2 IHC of CAIX

IHC was performed on previously constructed TMAs (*n* = 191) with three cores (0.6 mm) per patient to account for tumour heterogeneity. Specimens were dewaxed in Histo‐Clear and rehydrated through a decreasing gradient of ethanol. Heat‐induced antigen retrieval was carried out under pressure in a microwave using citrate buffer (pH 6), after which the sections were incubated in 3% H_2_O_2_. Non‐specific binding was blocked with 10% casein before overnight incubation with an anti‐CAIX antibody at 4°C (Bioscience, Slovakia, 1:500). TMAs were incubated in ImmPRESS and visualised with the DAB chromogen substrate (Vector Laboratories Inc., California, USA). Tissues were then counterstained in Harris haematoxylin (Thermo Fisher) before being dehydrated in ethanol and mounted with DPX (06522, Sigma‐Aldrich, St Louis, USA). Appropriate negative controls were included.

### 2.3 Scoring Methods

Stained TMA sections were scanned using a Hamamatsu and visualised in SlidePath (Version 4.0.9, Leica Biosystems, Newcastle, UK). The weighted histoscore method was used to score cytoplasmic and membranous CAIX expression as follows: (0× unstained cells) + (1× weakly stained cells) + (2× moderately stained cells) + (3× strongly stained cells). A range of scores from 0 to 300 were obtained. The raw histoscore count for CAIX staining is shown in Table S1. All three cores were scored separately, and an average score was taken. 10% of cores were double‐scored by an independent observer with a correlation coefficient of > 0.7.

Previous work from our group has stained the same ER− cohort with IHC for the lymphatic endothelial marker D2‐40 and Factor VIII to identify lymphatic and blood vessel invasion [20] and for CD68 and CD8 markers to assess the inflammatory cell infiltrate [21]. The cohort was also stained with haematoxylin and eosin to assess the tumour stroma percentage [22].

### 2.4 Statistical Analysis

Survminer and maxstat packages in R Studio (R Studio, Boston, MA, USA) were utilised to determine optimal thresholds for low and high CAIX expression groups for weighted histoscores in each cellular compartment based on overall survival (OS). Thresholds of 18 and 30 were generated based on the histoscore of cytoplasmic and membranous CAIX, respectively. Patients were grouped according to the weighted histoscore; those who scored more than 18 were classified as having high cytoplasmic CAIX expression, those who scored lower than or equal to 18 were classified as having low cytoplasmic CAIX, those who scored more than 30 were classified as having high membranous CAIX expression, and those who scored lower than or equal to 30 were classified as having low membranous CAIX.

The statistical analysis was performed using IBM SPSS statistics version 27. Patient survival was assessed by Kaplan–Meier analysis and log‐rank to test the significance. Univariate and multivariate Cox hazard regression was performed to estimate hazard ratios (HRs) and 95% confidence intervals (CIs). Chi‐squared testing was also utilised to determine an association, and the statistical difference was set at *p* < 0.05. Clinical outcomes measured were recurrence free survival (RFS), disease‐free survival (DFS), and OS.

### 2.5 Transcriptomic Analysis Using TempO‐Seq

Single tissue sections from ER− BC patients were excised and determined for TempO‐Seq analysis (*n* = 37) using a whole transcriptome panel according to the manufacturer's instructions. Briefly, formalin‐fixed paraffin‐embedded (FFPE) tissue was deparaffinised by heating before tissue digestion. The tissue lysate was then combined with a mixture of detector oligos (DOs), designed as pairs that anneal adjacent to one another on the target RNAs [23]. After a hybridisation step, unbound DOs were degraded, and the bound DOs were ligated into a complete probe sequence. The ligated probes were amplified in a PCR step using a unique primer set for each sample, introducing a sample‐specific barcode and Illumina adaptors (Figure S1). Barcoded samples were pooled into a single library and run on an Illumina HiSeq 2500 High Output v4 flowcell. Sequencing reads were demultiplexed using BCL2FASTQ software (Illumina, USA). FASTQ files were aligned to the Human Whole Transcriptome v2.0 panel, which consists of 22,537 probes, using STAR [24]. Up to two mismatches were allowed in the 50‐nucleotide sequencing read.

Raw gene count data were normalised and differentially expressed gene (DEG) analysis was carried out using the DESeq2 package (v1.30.0) [25] in R Studio (2020) (RStudio: Integrated Development for R. RStudio, PBC, Boston, MA). DEGs were visualised using volcano plots and MA plots. Significance was set to the adjusted *p*‐value (*p*adj) < 0.10 and the log2‐fold change (log2 FC) of > ± 1. Principal component analysis (PCA) was carried out to identify any clustering of high and low cytoplasmic CAIX expression. The heatmap was performed using ComplexHeatmap in R Studio to visualise the patterns of gene expression for the top 20 most significant DEGs.

### 2.6 Protein–Protein Interaction and Pathway Enrichment Analysis

Protein–protein interaction (PPI) networks were constructed using STRING (search tool for retrieval of interacting genes) database version 11.5 [26], which integrates both known and predicted PPIs, to predict functional interactions of proteins. One or more proteins can be searched at once using STRING, and the search can also be limited to the desired species “*Homo sapiens*”. The maximum number of interactors to show the first shell was limited to no more than 10 interactions. An interaction score > 0.4 (medium confidence), a false discovery rate (FDR) < 0.05, and a PPI enrichment *p*‐value < 0.05 were applied to construct PPI networks [27].

STRING database and Gene Ontology (GO) were utilised to identify pathways associated with DEGs in the high cytoplasmic CAIX expression group.

### 2.7 Real‐Time Quantitative PCR for Validation of Gene Expression

To further validate the stability of the seven selected genes (SERHL2, GALNT6, MUCL1, MMP7, PITX2, EACAM6 and SPNS2), real‐time quantitative PCR (RT‐qPCR) was performed on normoxic and hypoxic samples in MDA‐MB‐231 cells.

#### 2.7.1 Cell Cultures and Hypoxia Treatment

The human tumour cell lines MDA‐MB‐231 were cultured in Dulbecco's modified Eagle medium (DMEM) supplemented with 10% fetal bovine serum (FBS). The cells were incubated at 37°C in a 5% CO_2_ humidified incubator. Then, the cells were seeded into a 6‐well plate 24 h prior to experiment. For hypoxia experiments, the cells were maintained in an incubator chamber containing 1% oxygen for 4, 8, 16, 24, and 48 h; comparable normoxic samples collected at the same time points were used as control.

#### 2.7.2 RNA Extraction, Purification, Quantification and cDNA Conversion

MDA‐MB‐231 cells cultured under normoxic or hypoxic conditions were trypsinised and then collected by centrifugation at 12,000 g for 15 min at 4°C. Total cellular RNA was extracted using the TRIzol reagent (ambion). RNA concentration and purity were determined through 260/280 nm absorbance measures [28] using the NanoDrop spectrophotometer 2000 (Thermo Scientific). One microgram of total RNA was reverse transcribed into cDNA.

#### 2.7.3 Real‐Time Quantitative PCR

RT‐qPCR was performed with SYBR Green Master Mix (Thermo Fischer Scientific, Massachusetts, United States) using the Quant Studio 7 Flex Real‐Time PCR System (Applied Biosystems, Massachusetts, United States). The primer sequences and details of the product size and location are listed in Table S2. The ratio of target to GAPDH was calculated as ΔCt (delta cycle threshold) = Ct (target) − Ct (GAPDH), ratio (target) = 2 (^−ΔCt^). Bar charts showing the expression levels of genes were plotted using GraphPad Prism version 10 (GraphPad Software Inc.).

## 3 Results

### 3.1 ER− Cohort

#### 3.1.1 Clinicopathological Parameters

Of the 191 ER− patients, the majority (119, 62%) were over 50 years of age, had small tumours (≤ 20 mm, 51%), which were grade III (79%), and had negative lymph nodes (51%). The majority (124, 66%) of patients had TNBC and 57 (30%) had Her‐2 tumours. 127 patients (67%) received adjuvant chemotherapy, and 106 (56%) received adjuvant radiotherapy (Table S3). The schematic diagram of the proposed model based on the findings is shown in Figure S2.

#### 3.1.2 IHC of CAIX

**FIGURE 2:**
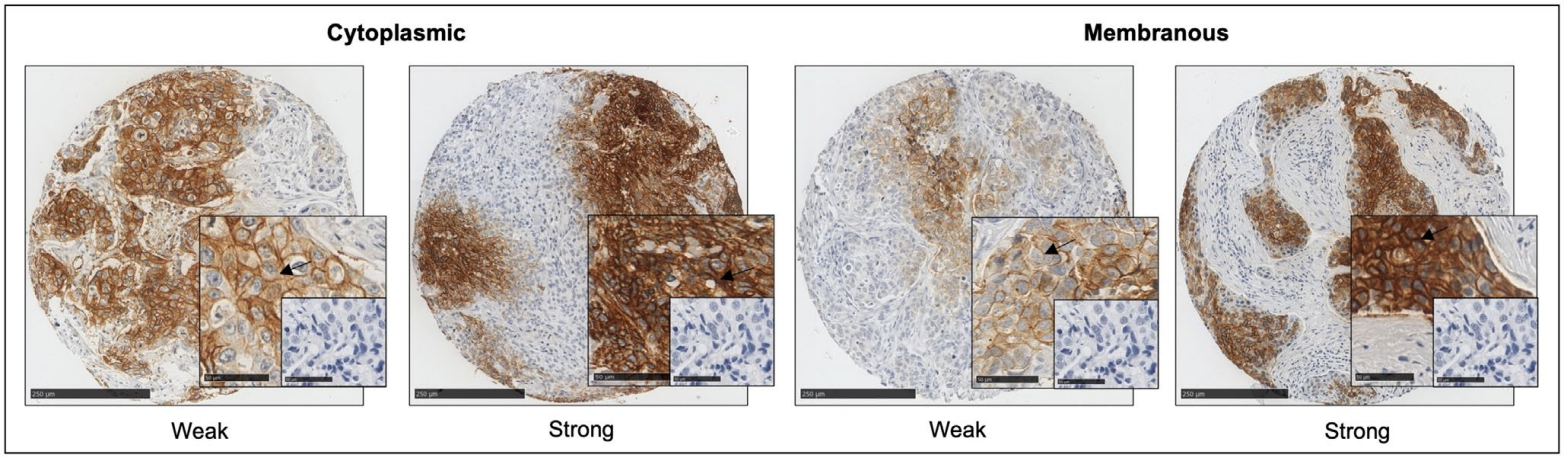
Immunohistochemical staining of CAIX in ER‐negative breast cancer patient samples. Weak and strong cytoplasmic and membranous CAIX expression in ER‐negative breast cancer TMAs. The small box shows negative control staining without an antibody. Scale bar 250 μm (large images) and 50 μm (small images).

After IHC was performed, cytoplasmic and membranous CAIX expression was observed, and a weighted histoscore was employed to quantify protein expression (Figure 2). The ICCC value for observers was 0.986 for cytoplasmic and 0.987 for membranous CAIX expression. Based on the R threshold, 60 patients had high cytoplasmic expression and 127 patients had low cytoplasmic expression. 59 patients had high membranous expression and 128 patients had low membranous expression.

#### 3.1.3 CAIX Protein Expression is Associated With Patient Survival and Clinicopathological Factors

**FIGURE 3:**
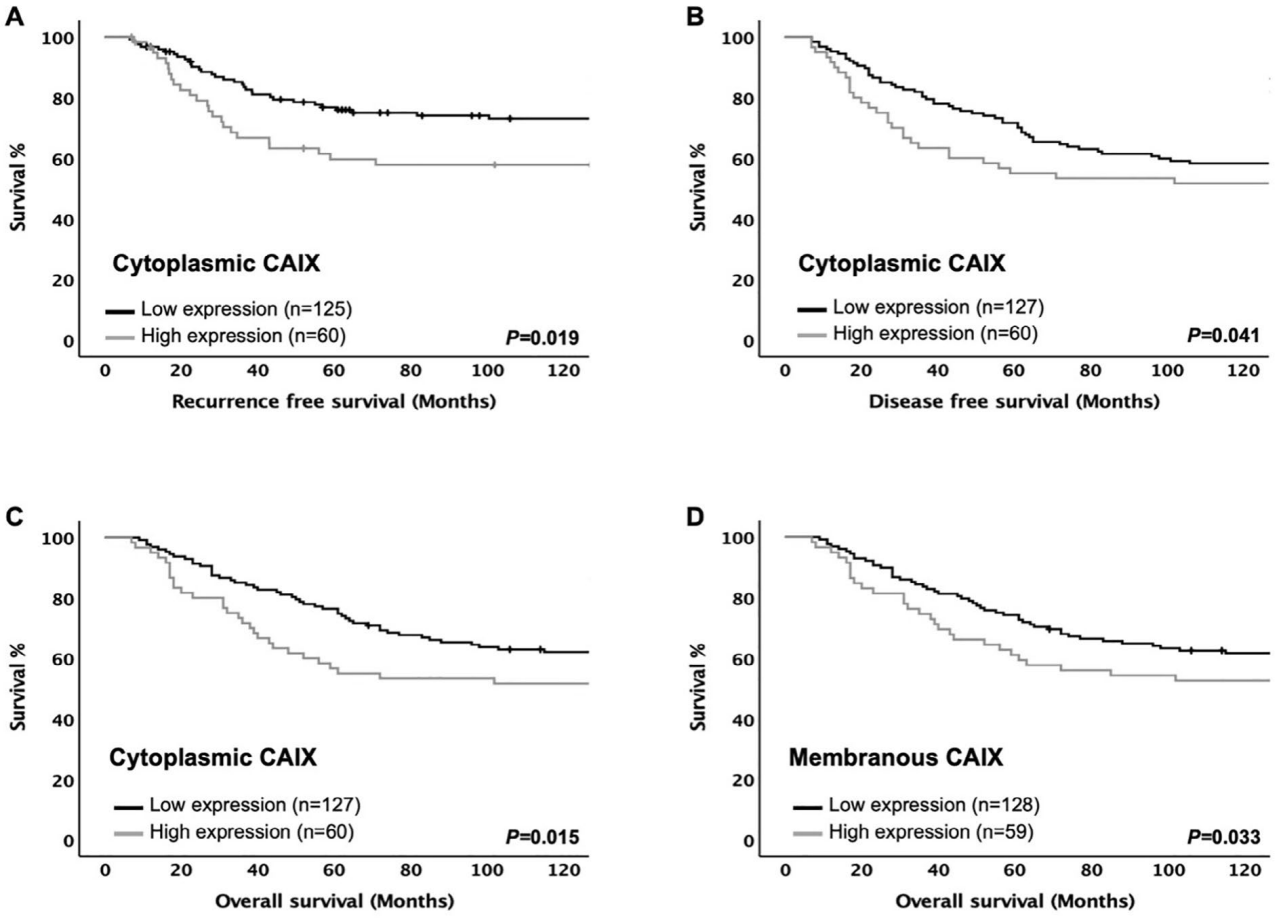
Expression of the CAIX protein and clinical outcome in ER‐negative cohort. Kaplan–Meier survival analysis based on cytoplasmic CAIX expression for recurrence free survival (A), disease‐free survival (B), overall survival (C), and membranous CAIX expression for overall survival (D).

To study the prognostic role of CAIX in ER− BC, Kaplan Meier survival analysis was employed. High cytoplasmic CAIX expression was significantly associated with shorter RFS (*p* = 0.019), DFS (*p* = 0.041), and OS (*p* = 0.015) (Figure 3A–C, respectively). Similarly, patients with a high membranous CAIX expression were observed to have shorter OS as compared with those who had a low expression (*p* = 0.033) (Figure 3D). CAIX was then entered into multivariate analysis, and it was an independent prognostic marker for RFS (HR = 2.34, 95% CI: 1.15–4.76, *p* = 0.019), DFS (HR = 1.99, 95% CI: 1.07–3.71, *p* = 0.029), and OS (HR = 2.45, 95% CI: 1.28–4.67, *p* = 0.007) (Tables 1, 2, 3, respectively). Similar results were observed with membranous CAIX expression, which was independently associated with OS (HR = 2.51, 95% CI: 1.28–4.94, *p* = 0.008) (Table 4).

**TABLE 1:** Univariate and multivariate analysis for recurrence free survival of cytoplasmic CAIX protein expression and clinicopathological characteristics in ER‐negative cohort (*n* = 191).

Clinicopathological characteristics	Univariate analysis		Multivariate analysis	
	HR (95% CI)	*p*	HR (95% CI)	*p*
Age (≤ 50/> 50 years)	0.98 (0.58–1.66)	0.943	—	—
Tumour size (mm) (≤ 20/21–50/> 50)	2.25 (1.45–3.51)	< 0.001*	1.67 (0.84–3.33)	0.146
Grade (I/II/III)	1.62 (0.87–3.02)	0.131	—	—
Involved lymph node (negative/positive)	2.84 (1.64–4.91)	< 0.001*	2.17 (0.94–5.01)	0.071
PR status (negative/positive)	0.43 (0.06–3.08)	0.397	—	—
Her‐2 status (negative/positive)	1.20 (0.69–2.07)	0.508	—	—
Ki67 index (low/high)	1.04 (0.59–1.81)	0.903	—	—
Lymphatic vessel invasion (no/yes)	4.04 (2.07–7.89)	< 0.001*	2.28 (1.00–5.18)	0.049*
Blood vessel invasion (no/yes)	2.76 (1.29–5.88)	0.009*	1.21 (0.49–2.99)	0.683
Tumour necrosis (low/high)	3.35 (1.34–8.38)	0.010*	8.99 (1.21–66.63)	0.032*
Klintrup–Mäkinen grade (low/high)	0.80 (0.56–1.15)	0.228	—	—
CD68^+^ (low/moderate/high)	0.69 (0.47–1.03)	0.071	—	—
CD8^+^ (low/moderate/high)	0.59 (0.39–0.86)	0.007*	0.83 (0.53–1.30)	0.410
CD138^+^ (low/moderate/high)	1.31 (0.90–1.89)	0.159	—	—
Tumour stroma percentage (low/high)	2.43 (1.45–4.07)	< 0.001*	3.63 (1.75–7.55)	< 0.001*
Tumour budding (low/high)	2.53 (1.49–4.31)	< 0.001*	1.09 (0.46–2.55)	0.846
Adjuvant chemotherapy (no/yes)	1.40 (0.79–2.49)	0.249	—	—
Adjuvant radiotherapy (no/yes)	1.50 (0.88–2.57)	0.136	—	—
Cytoplasmic CAIX (low/high)	1.84 (1.09–3.09)	0.019*	2.34 (1.15–4.76)	0.019*

Abbreviations: Lum A, Luminal A; Lum B, Luminal B.

* *p* < 0.05.

**TABLE 2:** Univariate and multivariate analysis for disease‐free survival of cytoplasmic CAIX protein expression and clinicopathological characteristics in ER‐negative cohort (*n* = 191).

Clinicopathological characteristics	Univariate analysis		Multivariate analysis	
	HR (95% CI)	*p*	HR (95% CI)	*p*
Age (≤ 50/> 50 years)	1.46 (0.95–2.25)	0.088	—	—
Tumour size (mm) (≤ 20/21–50/> 50)	1.84 (1.29–2.61)	< 0.001*	1.77 (1.01–3.09)	0.045*
Grade (I/II/III)	1.09 (0.72–1.65)	0.671	—	—
Involved lymph node (negative/positive)	2.13 (1.41–3.21)	< 0.001*	1.46 (0.72–2.94)	0.296
PR status (negative/positive)	0.51 (0.13–2.07)	0.344	—	—
Her‐2 status (negative/positive)	1.25 (0.82–1.91)	0.309	—	—
Ki67 index (low/high)	0.97 (0.62–1.51)	0.875	—	—
Lymphatic vessel invasion (no/yes)	3.18 (1.87–5.41)	< 0.001*	3.04 (1.71–5.40)	< 0.001*
Blood vessel invasion (no/yes)	2.92 (1.58–5.41)	< 0.001*	1.76 (0.86–3.63)	0.123
Tumour necrosis (low/high)	1.75 (1.00–3.04)	0.048*	2.09 (0.85–5.14)	0.110
Klintrup–Mäkinen grade (low/high)	0.86 (0.65–1.14)	0.291	—	—
CD68^+^ (low/moderate/high)	0.74 (0.54–1.02)	0.066	—	—
CD8^+^ (low/moderate/high)	0.62 (0.45–0.85)	0.003*	0.73 (0.50–1.05)	0.089
CD138^+^ (low/moderate/high)	1.28 (0.95–1.72)	0.111	—	—
Tumour stroma percentage (low/high)	1.96 (1.30–2.95)	0.001*	2.46 (1.21–4.99)	0.013*
Tumour budding (low/high)	1.97 (1.27–3.06)	0.002*	1.19 (0.61–2.29)	0.611
Adjuvant chemotherapy (no/yes)	0.89 (0.59–1.37)	0.622	—	—
Adjuvant radiotherapy (no/yes)	1.16 (0.77–1.75)	0.480	—	—
Cytoplasmic CAIX (low/high)	1.54 (1.01–2.33)	0.041*	1.99 (1.07–3.71)	0.029*

*Statistically significant *p* value < 0.05.

**TABLE 3:** Univariate and multivariate analysis for overall survival of cytoplasmic CAIX protein expression and clinicopathological characteristics in ER‐negative cohort (*n* = 191).

Clinicopathological characteristics	Univariate analysis		Multivariate analysis	
	HR (95% CI)	*p*	HR (95% CI)	*p*
Age (≤ 50/> 50 years)	1.69 (1.06–2.67)	0.026*	1.92 (1.01–3.67)	0.047*
Tumour size (mm) (≤ 20/21–50/> 50)	1.82 (1.26–2.63)	0.001*	1.46 (0.77–2.77)	0.247
Grade (I/II/III)	1.11 (0.72–1.70)	0.651	—	—
Involved lymph node (negative/positive)	2.13 (1.38–3.27)	< 0.001*	1.97 (0.89–4.35)	0.094
PR status (negative/positive)	0.49 (0.12–2.03)	0.332	—	—
Her—2 status (negative/positive)	1.06 (0.67–1.66)	0.807	—	—
Ki67 index (low/high)	1.04 (0.66–1.65)	0.871	—	—
Lymphatic vessel invasion (no/yes)	3.29 (1.88–5.77)	< 0.001*	2.17 (1.01–4.65)	0.046*
Blood vessel invasion (no/yes)	3.38 (1.81–6.31)	< 0.001*	3.02 (1.45–6.29)	0.003*
Tumour necrosis (low/high)	1.67 (0.94–2.97)	0.079	—	—
Klintrup‐Mäkinen grade (low/high)	0.85 (0.64–1.14)	0.275	—	—
CD68^+^ (low/moderate/high)	0.67 (0.48–0.94)	0.022*	0.66 (0.45–0.97)	0.034*
CD8^+^ (low/moderate/high)	0.59 (0.42–0.82)	0.002*	0.75 (0.48–1.15)	0.182
CD138^+^ (low/moderate/high)	1.26 (0.92–1.72)	0.151	—	—
Tumour stroma percentage (low/high)	1.81 (1.18–2.77)	0.006*	2.41 (1.27–4.58)	0.007*
Tumour budding (low/high)	2.01 (1.28–3.18)	0.003*	0.91 (0.44–1.87)	0.801
Adjuvant chemotherapy (no/yes)	0.84 (0.54–1.29)	0.424	—	—
Adjuvant radiotherapy (no/yes)	1.06 (0.69–1.62)	0.804	—	—
Cytoplasmic CAIX (low/high)	1.69 (1.09–2.59)	0.015*	2.45 (1.28–4.67)	0.007*

*Statistically significant *p* value < 0.05.

**TABLE 4:** Univariate and multivariate analysis for overall survival of membranous CAIX protein expression and clinicopathological characteristics in ER‐negative cohort (*n* = 191).

Clinicopathological characteristics	Univariate analysis		Multivariate analysis	
	HR (95% CI)	*p*	HR (95% CI)	*p*
Age (≤ 50/> 50 years)	1.69 (1.06–2.67)	0.026*	1.92 (1.01–3.65)	0.046*
Tumour size (mm) (≤ 20/21–50/> 50)	1.82 (1.26–2.63)	0.001*	1.44 (0.77–2.69)	0.255
Grade (I/II/III)	1.11 (0.72–1.70)	0.651	—	—
Involved lymph node (negative/positive)	2.13 (1.38–3.27)	< 0.001*	1.78 (0.80–3.95)	0.155
PR status (negative/positive)	0.49 (0.12–2.03)	0.332	—	—
Her‐2 status (negative/positive)	1.06 (0.67–1.66)	0.807	—	—
Ki67 index (low/high)	1.04 (0.66–1.65)	0.871	—	—
Lymphatic vessel invasion (no/yes)	3.29 (1.88–5.77)	< 0.001*	3.37 (1.76–6.45)	< 0.001*
Blood vessel invasion (no/yes)	3.38 (1.81–6.31)	< 0.001*	3.44 (1.64–7.18)	0.001*
Tumour necrosis (low/high)	1.67 (0.94–2.97)	0.079	—	—
Klintrup–Mäkinen grade (low/high)	0.85 (0.64–1.14)	0.275	—	—
CD68^+^ (low/moderate/high)	0.67 (0.48–0.94)	0.022*	0.66 (0.45–0.99)	0.044*
CD8^+^ (low/moderate/high)	0.59 (0.42–0.82)	0.002*	0.59 (0.42–0.86)	0.005*
CD138^+^ (low/moderate/high)	1.26 (0.92–1.72)	0.151	—	—
Tumour stroma percentage (low/high)	1.81 (1.18–2.77)	0.006*	1.74 (0.82–3.67)	0.148
Tumour budding (low/high)	2.01 (1.28–3.18)	0.003*	0.91 (0.43–1.91)	0.803
Adjuvant chemotherapy (no/yes)	0.84 (0.54–1.29)	0.424	—	—
Adjuvant radiotherapy (no/yes)	1.06 (0.69–1.62)	0.804	—	—
Membranous CAIX (low/high)	1.69 (1.09–2.59)	0.015*	2.51 (1.28–4.94)	0.008*

*Statistically significant *p* value < 0.05.

The correlation between cytoplasmic CAIX and the clinical characteristics of ER− patients is shown in Table S4. The chi‐square test showed a significant association between high cytoplasmic CAIX expression and tumour necrosis (*p* = 0.003).

#### 3.1.4 Gene Expression From TempO‐Seq Data

To further investigate the prognostic relevance of ER− BC patients with high and low cytoplasmic CAIX, transcriptomic data obtained from FFPE breast tissue in the same ER− cohort was utilised (*n* = 37). Clinicopathological characteristics of these patients are shown in Table S5. There was a significant association between high cytoplasmic CAIX protein expression and lymph node negativity (*p* = 0.023) (Table S6).

**FIGURE 4:**
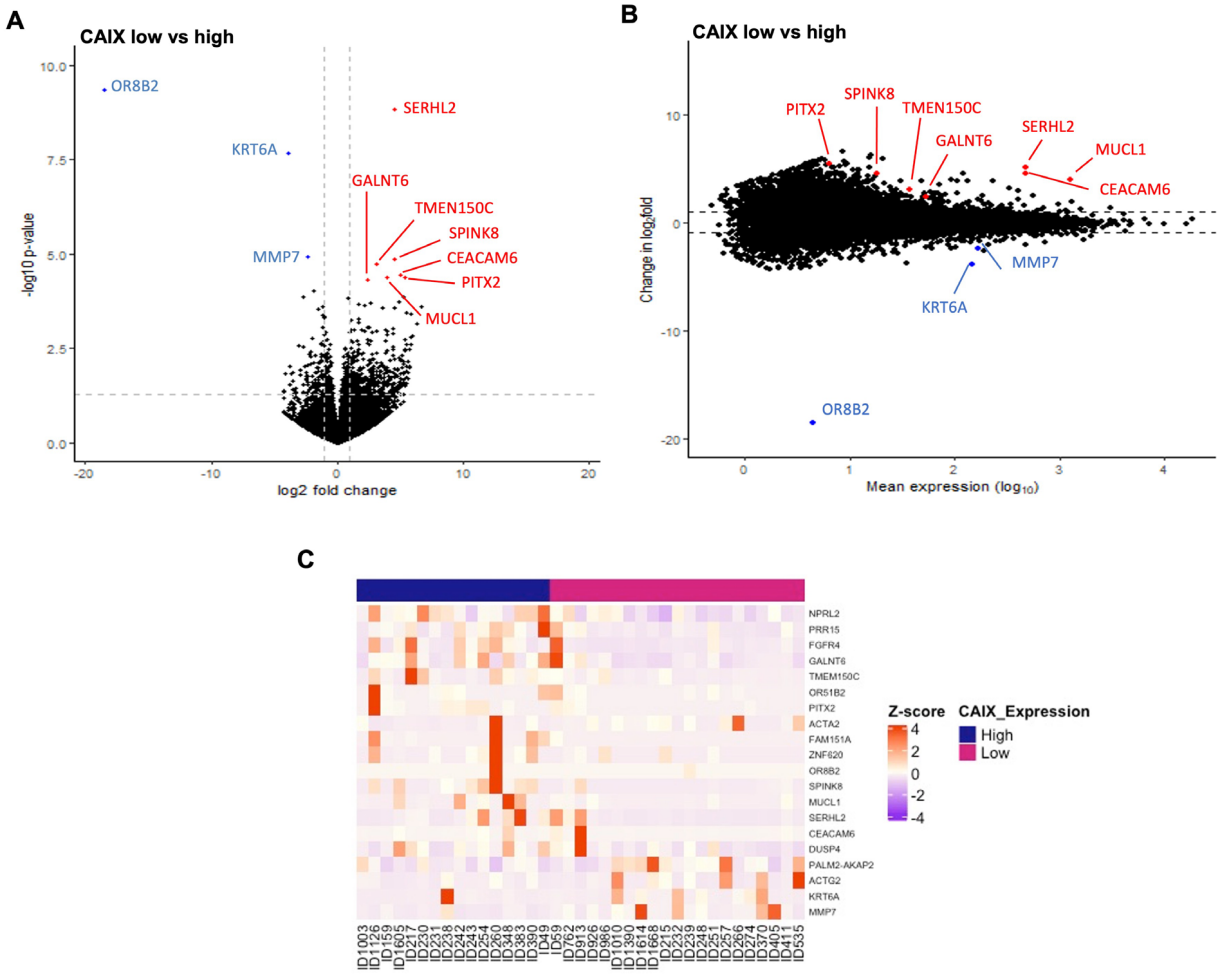
Differential expression gene analysis in ER‐negative cohort relative to cytoplasmic CAIX expression groups. (A) Volcano plot showing the distribution of gene expression fold changes and *p* values between patients with high and low cytoplasmic CAIX. (B) MA plot showing 10 DEGs comparing high and low cytoplasmic CAIX expression tumours. Red means upregulated and blue means downregulated genes. (C) Heatmap of the top 20 DEGs between low (pink) and high (blue) cytoplasmic CAIX protein expression.

Ten DEGs were identified when comparing tumour cases with high CAIX to those with low CAIX protein expression. Seven genes were upregulated (SERHL2, SPINK8, TMEM150C, CEACAM6, MUCL1, PITX2 and GALNT6), and three genes were downregulated (OR8B2, KRT6A and MMP7), as shown in the volcano plot and MA plot (Figure 4A,B, respectively).

The results showed no obvious classification between two groups, as illustrated by the PCA plot (Figure S3). There was a clear pattern in the gene expression profile between tumours with high and low CAIX expressions when the top 20 DEGs were considered, as shown in the heatmap (Figure 4C).

#### 3.1.5 PPI Network Construction

**FIGURE 5:**
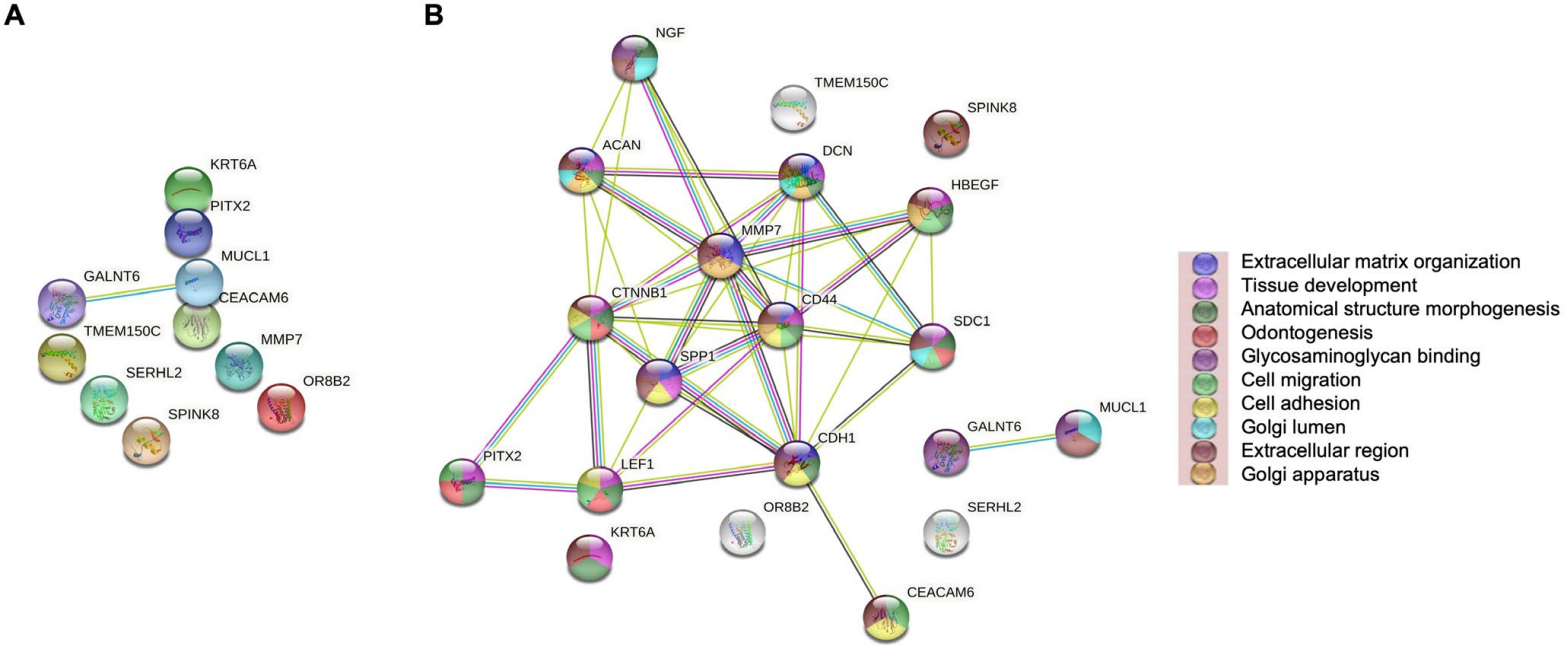
Protein–protein interaction of differential expression genes in ER‐negative cohort. STRING interaction network diagram showing relationships between DEGs from full transcriptional sequencing on a subset of the ER‐negative cohort, (A) PPI network analysis for 10 proteins, (B) PPI network analysis for extra added 10 proteins.

The interaction networks of DEGs with a significant *p*adj were constructed and visualised by the STRING database online tool. Only 2 of the 10 genes examined could be connected in a PPI network. There was significant interaction between MUCL1 and GALNT6 proteins. However, eight proteins did not have interactions with other proteins (Figure 5A). Ten more proteins have been added to the standard protein–protein association network in STRING, using the “more” button. Proteins will automatically appear in the network based on their known associations with host proteins, showing 20 nodes and 42 edges, with the PPI enrichment *p*‐value of 1.12e‐06 (Figure 5B). The network nodes are proteins, the edges represent the predicted functional associations, and the colour represents their response molecular function. However, there were three nodes, coloured white, whose function could not be identified on the STRING online database.

#### 3.1.6 Pathway Enrichment Analyses of DEGs

Signalling pathways associated with the identified DEGs in the high cytoplasmic CAIX expression group within ER− patients were obtained using the STRING database. CEACAM6 and MUCL1 upregulated genes were linked with Reactome pathways including extracellular matrix organisation, degradation of extracellular matrix and disease of glycosylation (Table S7).

GO was also performed to view the signalling pathways linked with DEGs in the high CAIX expression group within ER− cohort. The cnetplot plot showed five proteins (PITX2, TMEM150C, MMP7, GALNT6 and MUCL1) associated with significantly enriched gene sets (Figure S4). MUCL1 and GALNT6 genes were associated with protein O‐linked glycosylation and O‐glycan processing. MMP7 and TMEM150C genes were associated with the cellular response to the mechanical stimulus pathway. The PITX2 gene was associated with cardiac neural crest cell development involved in heart development.

### 3.2 Node‐Negative Subgroup

#### 3.2.1 Gene Expression From TempO‐Seq Data

**FIGURE 6:**
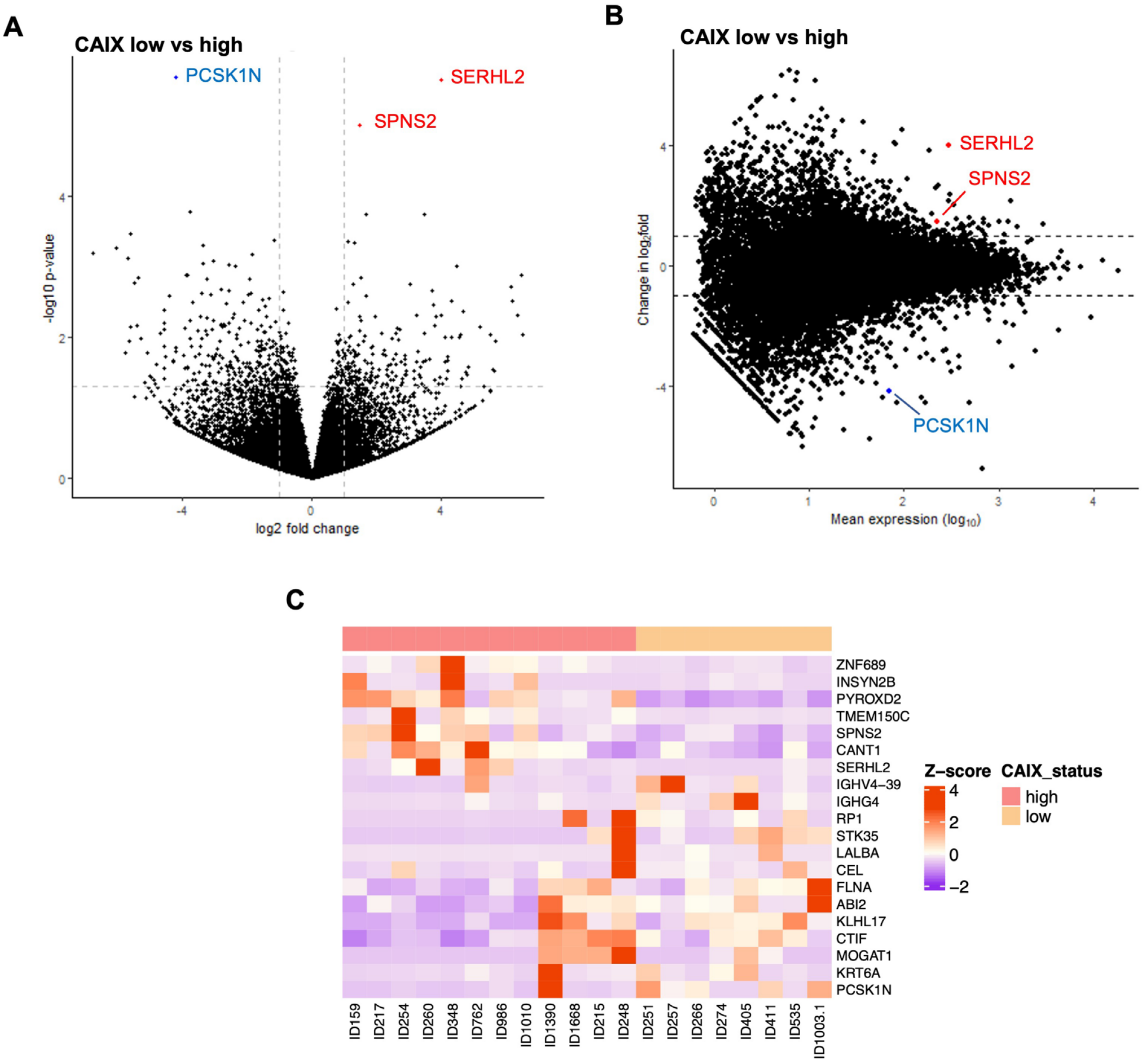
Differential expression genes analysis in lymph node‐negative patients relative to cytoplasmic CAIX expression groups. (A) Volcano plot showing the distribution of gene expression fold changes and *p*‐values between patients with high and low cytoplasmic CAIX. (B) MA plot showing 3 DEGs comparing high and low cytoplasmic CAIX expression tumours. Red means up‐regulated and blue means down‐regulated genes. (C) Heatmap of the top 20 DEGs between low (Pink) and high (beige) cytoplasmic CAIX protein expression.

High cytoplasmic CAIX expression is associated with negative lymph node status; therefore, the transcriptomic analysis in the node‐negative subgroup was performed. Three genes were significantly differentially expressed across the node‐negative subgroup. Two genes were upregulated (SERHL2 and SPNS2) in low cytoplasmic CAIX tumours, while one gene (PCSK1N) was downregulated, as shown in the volcano plot and MA plot (Figure 6A,B, respectively).

PCA revealed no clustering of gene expression between two cytoplasmic CAIX expression groups (Figure S5). A heatmap showed a clear pattern in the gene expression profile between tumours with low compared to high expression (Figure 6C).

#### 3.2.2 PPI Network Construction

**FIGURE 7:**
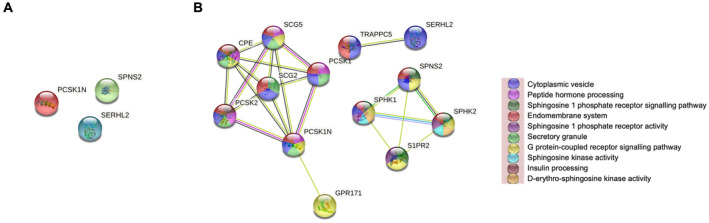
Protein–protein interaction of differential expression genes in lymph node‐negative patients. STRING interaction network diagram showing relationships between DEGs from full transcriptional sequencing on a subset of the node‐negative group: (A) PPI network analysis for three proteins and (B) PPI network analysis for extra added 10 proteins.

The proteins that correspond to the top 10 significant DEGs were used to show a network around the input proteins using the STRING online tool. There was no significant interaction among PCSK1N, SERHL2, and SPNS2 proteins within a PPI network (Figure 7A). To create a network around the input proteins, a total of 10 proteins were added. These 13 nodes had 22 edges, with a PPI enrichment *p*‐value of 0.0009 (Figure 7B).

#### 3.2.3 Pathway Enrichment Analyses of DEGs

To identify signalling pathways associated with DEGs in high cytoplasmic CAIX expression in the subgroup of node‐negative tumours, the STRING database was used. The SPNS2 gene was linked with the sphingolipid de novo biosynthesis pathway, as shown in Table S8. As the node‐negative data implicated that sphingosine kinase signalling was associated with hypoxia, the protein level was considered to investigate the effect of sphingosine kinase on the hypoxia pathway. Previous work from our group used IHC to look at the sphingosine kinase pathway in the same ER− patient cohort [29]. A chi‐squared analysis using these data in the node‐negative group showed no significant correlations between the sphingosine kinase pathway and CAIX protein expression. However, an association was found between cytoplasmic sphingosine kinase‐1 (SphK1) and HIF‐1α protein expression (Table 5).

**TABLE 5:** Correlations among SphK1, S1P4, CAIX and HIF‐1α protein expression in the node‐negative group.

Markers	Membranous SphK1	Cytoplasmic SphK1	Nuclear SphK1	Membranous S1P4	Cytoplasmic S1P4	Nuclear S1P4
Cytoplasmic CAIX	0.575	0.965	0.781	0.320	0.152	0.124
Cytoplasmic HIF‐1α	0.289	0.017*	0.328	0.415	0.992	0.397

Abbreviations: CAIX, carbonic anhydrase IX; HIF‐1α, hypoxia‐inducible factor‐1α; S1P4, sphingosine 1‐phosphate receptor 4; SphK1, sphingosine kinase 1. *Statistically significant *p* value < 0.05.

### 3.3 RT‐qPCR Validation of Gene Expression in the BC Cell Line

**FIGURE 8:**
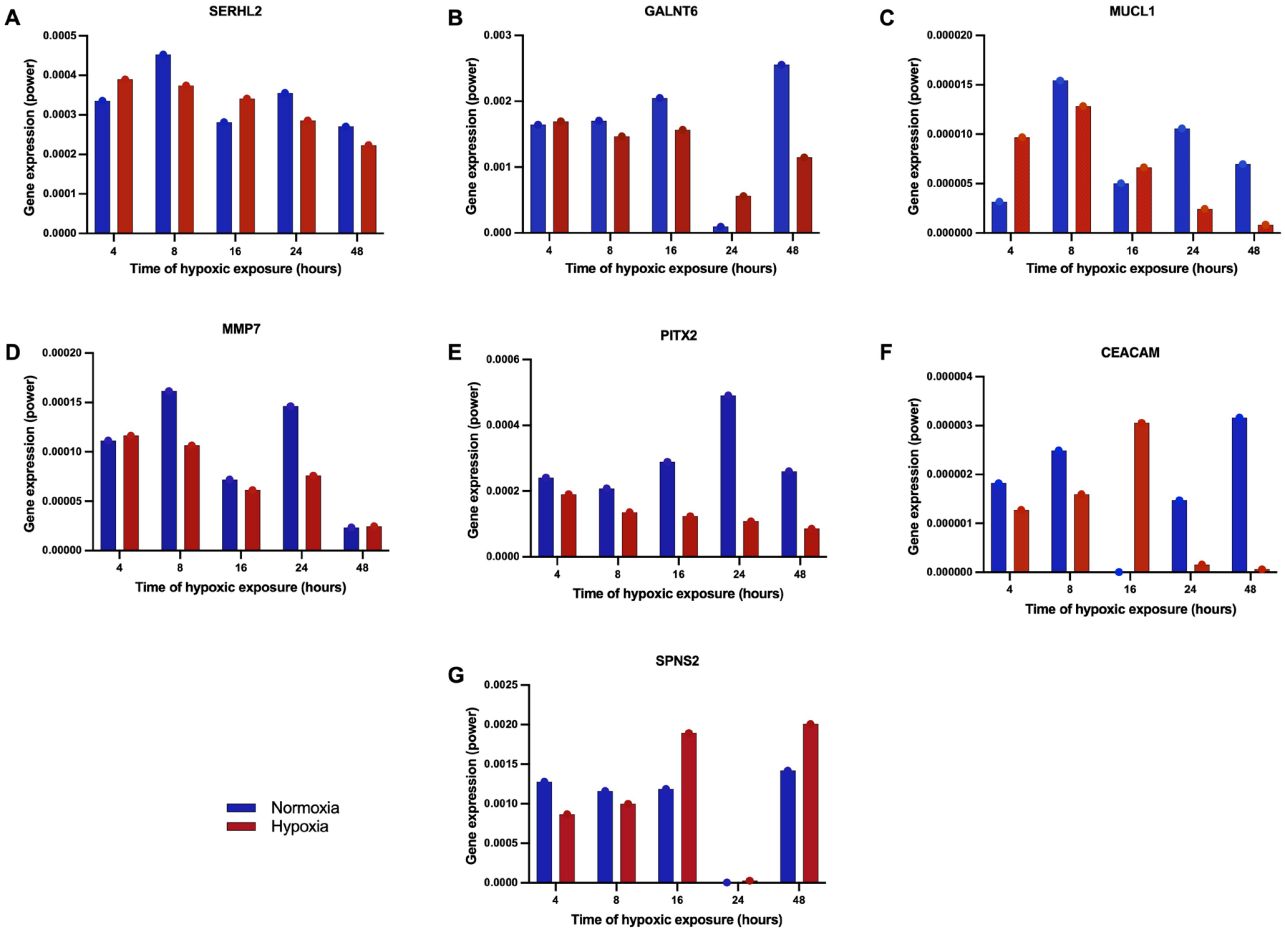
Differentially expressed genes validated by RT‐qPCR. Expression levels of genes in normoxic and hypoxic MDA‐MB‐231 cells at different time points, 4, 8, 16, 24, and 48 h: (A) SERHL2, (B) GALNT6, (C) MUCL1, (D) MMP7, (E) PITX2, (F) CEACAM6 and (G) SPNS2.

The RT‐qPCR array was established on hypoxic and normoxic MDA‐MB‐231 cells after different episodes of hypoxia (4, 8, 16, 24, and 48 h) to validate the expression of SERHL2, GALNT6, MUCL1, MMP7, PITX2, CEACAM6 and SPNS2. Variable expression levels of all the genes were observed in all the samples examined, as shown in bar charts (Figure 8A–G). Expressions of SERHL2, GALNT6, MUCL1, PITX2 and SPNS2 were upregulated across normoxic and hypoxic cell lines with a considerable decrease in MMP7 and CEACAM6 expression (Table S9). There was reduction in SERHL2 and PITX2 genes' expression with long‐term hypoxia (Figure 8A,E). A marked reduction in MUCL1 expression was observed after being exposed to eight hypoxic shots, and the highest induction was observed at 8 h incubation under hypoxic conditions (Figure 8C). After cell cultivation for 16 h in hypoxic conditions, the level of GALNT6 expression in cells slightly decreases (Figure 8B). However, our results revealed that longer exposure to hypoxia was associated with the increased expression of the SPNS2 gene (Figure 8G).

## 4 Discussion

In the present analysis, cytoplasmic CAIX expression was consistently associated with survival in ER− patients. In ER− tumours, most of the DEGs in the group with high cytoplasmic CAIX expression were upregulated; however, only a few of these genes attained statistical significance. Ten significant genes were identified, namely, OR8B2, SERHL2, KRT6A, MMP7, SPINK8, TMEM150C, CEACAM6, MUCL1, PITX2 and GALNT6, which warrant further investigation. In contrast, in those patients with node‐negative disease, only three genes were significantly differentially expressed, namely, PCSK1N, SERHL2, and SPNS2. Therefore, it would appear that different genes are differentially expressed in more advanced diseases and that only SERHL2 remained differentially expressed (a log2‐fold increase of approximately 4) with disease progression.

According to our findings, patients with high CAIX expression were significantly associated with poor survival, which was in concordance with previous studies [18, 19]. An explanation for the association between CAIX expression and poor prognosis may lie in the nature of its involvement in pH regulation in breast tissue, supporting BC cell survival [12]. In line with previous results, we have shown that high CAIX‐expressing tumours were associated with tumour necrosis [30], as a consequence of hypoxic occurrence, suggesting that CAIX was closely associated with indicators of an aggressive phenotype and poor prognosis [31]. The present results show that CAIX is functionally involved in several aspects of cancer growth and development in BC, and this appears to be particularly strong in ER− disease. In addition, our results showed that high CAIX‐expressing tumours were associated with lymph node negativity, which was consistent with a previous study in BC [32]. This finding suggests that levels of CAIX may be used to select high‐risk patients with negative lymph node status who would benefit from systemic adjuvant therapy.

SERHL2 (serine hydrolase‐like protein 2) belongs to the serine hydrolase family [33]. It was identified in TNBC for predicting the chemotherapeutic response [34]. However, from the literature, the present study is the first to document the association of SERHL2 with cancer hypoxia. Moreover, due to its function and the fact that it is consistently over‐expressed independent of the disease ER‐alpha stage, it may prove to be a useful therapeutic target.

In ER− patients, the result from the PPI networks of the tumours with the high expression of cytoplasmic CAIX demonstrated that two proteins, namely, GALNT6 and MUCL1, had significant interactions with each other (Figure 5A). GALNT6 (polypeptide N‐acetylgalactosaminyltransferase 6) was associated with poor prognosis in BC [35]. GALNT6 has been shown to promote tumorigenesis and metastasis by catalysing mucin‐type O‐glycosylation‐mediated stabilisation of MUCL1 in BC cells [36]. MUCL1 (Mucin‐like 1) is highly expressed in ER− BC [37] and promotes BC metastasis via promoting EMT [38]. Therefore, the present results confirm the association of GALNT6 and MUCL1 with more advanced stages in patients with ER− BC and suggest that hypoxia is a significant driver of GALNT6 expression in these patients. To our knowledge, the relationship between such expression of GALNT6 and MUCL1 and tumour hypoxia in ER− BC has not been previously documented and therefore requires confirmation in further studies.

The remaining DEG input proteins, MMP7, PITX2 and CEACAM6, were shown to interact with their STRING database partner proteins (Figure 5B). We found an inverse correlation between CAIX and MMP7 (matrix metallopeptidase 7) as being downregulated at the mRNA level. Contrarily, other studies reported that hypoxia promotes the expression of MMP7 and BC invasion [39]. Having said that, the cohort was much smaller with only 37 patients were used for TempO‐Seq analysis.

PITX2 (paired‐like homeodomain 2) serves as a predictive and prognostic biomarker in BC patients [40, 41]. However, to date, there have been no reports of the prognostic significance of PITX2 expression and cancer hypoxia, and the present study is the first to document the association of PITX2 with tumour hypoxia. CEACAM6 (carcinoembryonic antigen cell adhesion molecule 6) is significantly upregulated in oestrogen‐deprived BC cells [42], associated with BC progression [43] and poor prognosis [44]. However, the association of CEACAM6 with tumour hypoxia is yet to be explained, and the present study is the first to document the association of CEACAM6 with cancer hypoxia.

GO and STRING database demonstrated that MUCL1 and GALNT6 genes were linked with reactome pathways including extracellular matrix organisation, degradation of extracellular matrix and disease of glycosylation, which are known to play a direct role in the progression of BC [45, 46].

Clinically, nodal status remains an important prognostic factor; therefore, gene expression analysis was compared between the whole cohort and those node‐negative patients. With reference to the node‐negative tumours, the STRING online method demonstrated no significant interaction between expressed proteins. SPNS2 showed interaction with its partner proteins, which were added from the STRING online database including SphK1, SphK2, and S1PR2. These findings identify a number of interactions and associations between these proteins that influence BC progression.

SPNS2 (sphingolipid transporter 2) controls sphingosine 1‐phosphate (S1P) release and modifies S1P activity as an S1P transporter [47]. S1P is a sphingosine‐derived lipid mediator that is catalysed by two sphingosine kinases (SphK1 and SphK2) [47]. S1P can act as a ligand on a family of five S1P‐specific G protein‐coupled receptors (S1P_1–5_) or be exported from cells via SPNS2 or binds to particular intracellular target proteins [48]. High S1P expression in BC was associated with lymphatic metastasis by affecting tumour microenvironment [49]. SphK1 mRNA promotes TNBC cell metastasis and invasion [50] and is associated with poor survival in ER+ and ER− BC [51, 52]. S1P receptors contribute to cancer progression by enhancing the proliferation of ER+ and ER− BC cells [53].

In the present study, the STRING online method showed the SPNS2‐associated sphingolipid de novo biosynthesis pathway. SPNS2 promoted cancer genesis, apoptosis and migration via S1P/S1PRs pathways that activated downstream signalling such as STAT3, AKT, ERK, Ras and Rac [54]. In ER− BC cells, S1P binding to S1P4 stimulates activation of the ERK1/2 pathway and correlates with poor prognosis [29]. Furthermore, through S1PR3‐mediated upregulation of the notch intracellular domain, SphK1 stimulates BC metastasis [55]. Inhibition of SphK1 results in cell death in human BC cells [56], indicating that tumour SphK1/S1P signalling plays a vital role in growth/proliferation. However, such data are hard to interpret and therefore it is important to validate at the protein level. SPNS2 was validated by IHC in our lab [29]. For a given gene, at the protein level, a statistically significant correlation between cytoplasmic HIF‐1α and cytoplasmic SphK1 protein expression was observed in the node‐negative group (*p* = 0.017). These findings are in line with previous in vitro experiments [57], suggesting that SphK1 acts as a modulator of HIF‐1α. Studies have shown that the transcriptional regulation of SphK1 has been influenced by both HIF‐1α and HIF‐2α and that the SphK1 promoter contains two hypoxia‐inducible factor‐responsive elements [58]. SphK1/S1P signalling has also been linked to the regulation of HIF‐2α expression, which can drive aggressive tumours; therefore, knockdown of SphK1/S1P is associated with lower HIF‐2α protein expression [59]. However, the absence of correlation between SphK1 and CAIX could lie in the methodology of IHC, as well as in the primary antibodies used.

The present study demonstrated that although a variety of genes were expressed in hypoxia mediated by cytoplasmic CAIX in ER− cohort, only three genes were expressed in the node‐negative group. This finding supports the idea that apparent differences in DEGs between two patient groups could be required to include a representation of specific pathways that might be involved in BC progression. In fact, SPNS2 has superior performance compared with other DEGs.

In the present study, the expression of the seven validated genes (SERHL2, GALNT6, MUCL1, MMP7, PITX2, CEACAM6 and SPNS2) was achieved in MDA‐MB‐231 cells lines at multiple time points (4, 8, 16, 24, and 48 h). Among the seven genes, five (SERHL2, GALNT6, MUCL1, PITX2 and SPNS2) were consistently upregulated, while the remaining two genes (MMP7 and CEACAM6) were downregulated. Importantly, among the five upregulated genes, SPNS2 expression increased over time in hypoxia. The SPNS2 pathway was dependent on HIF activity. Hypoxia increases the production and release of S1P in glioma cells [60], upregulates SphK1 that promotes the migration of endothelial cells [60], stimulates SphK2 expression and S1P release in adenocarcinoma cells [61], and stimulates SphK1 and SphK2 expression in pulmonary smooth muscle cells [62]. Although the expression of two genes (MMP7 and CEACAM6) were low, the response in only one cell line cannot completely represent the complex and dynamic responses in patients, which explains why some q‐PCR results are different from the RNASeq dataset.

Finally, the genes identified in these analyses provide important data for future pathway research, with the potential to identify new targets that could lead to better treatment and a deeper understanding of the genes involved in tumour progression and metastasis. The current work has provided further evidence that co‐expression of HIF‐1α and SphK1 in this cohort of patients with node‐negative BC may rationally support the use of medicines targeting the HIF molecular cascade as a novel target for drug development.

Limitations of the present study include a limited sample size and perhaps selection bias in the patients analysed (pathology diagnostic archive), which may limit the representativeness of the samples and thus an increase in the risk of bias. Therefore, further studies are required to confirm the present results. In particular, the unique observation that SERHL2 was differentially expressed requires confirmation in other studies. In the present study 10% of cores were co‐scored by a second observer with an interclass correlation coefficient (ICCC) > 0.7. Therefore, it was unlikely that a significant observer error was introduced in the present study. Also, old FFPE samples could affect RNA studies, so further studies are required within new FFPE samples to confirm the result.

## 5 Conclusion

In the present study, cytoplasmic CAIX was an independent prognostic factor for RFS, DFS, and OS in ER− BC. The transcriptomic data identified 10 genes significantly associated with CAIX tumours in ER− cohort. However, due to the heterogeneous population, subsequent analysis of lymph node‐negative patients was performed with SPNS2 being of particular interest. This gene profile was confirmed at the protein level, and it was very useful. It could provide a strong tool for identifying subgroups of patients with node‐negative BC who are most likely to react to hypoxic tumour therapy, reducing over‐treatment in substantial numbers of patients. If verified in larger cohorts, this prognostic signature could guide the recommendation of hypoxia‐focused therapy in patients with lymph node‐negative primary BC.

## Author Contributions

**Suad A. K. Shamis:** data curation (equal), formal analysis (equal), investigation (equal), methodology (equal), software (equal), visualization (equal), writing – original draft (equal). **Jean Quinn:** methodology (equal), writing – review and editing (equal). **Sara Al‐Badran:** methodology (equal), writing – review and editing (equal). **Molly McKenzie:** formal analysis (equal), writing – review and editing (equal). **Phimmada Hatthakarnkul:** formal analysis (equal), investigation (equal), software (equal), visualization (equal), writing – review and editing (equal). **Gerard Lynch:** formal analysis (equal), investigation (equal), software (equal), visualization (equal), writing – review and editing (equal). **Guang‐Yu Lian:** formal analysis (equal), methodology (equal), software (equal), validation (equal), writing – review and editing (equal). **Warapan Numprasit:** formal analysis (equal), methodology (equal), validation (equal), writing – review and editing (equal). **Laszlo Romics Jr.:** resources (equal), writing – review and editing (equal). **Ditte Andersen:** data curation (equal), methodology (equal), writing – review and editing (equal). **Elizabeth Mallon:** resources (equal), writing – review and editing (equal). **Donald C. McMillan:** conceptualization (equal), project administration (equal), resources (equal), supervision (equal), writing – review and editing (equal). **Joanne Edwards:** conceptualization (equal), project administration (equal), resources (equal), supervision (equal), writing – review and editing (equal).

## Ethics Statement

This study was approved by the West Glasgow University Hospitals Research Ethics Committee (REC: 16/WS/0207), in accordance with Human Tissue (Scotland) Act 2006.

## Consent

The material used in this study was obtained from the Pathology Diagnostic Archive. These samples were surplus material, not collected for project specific purpose. In accordance with HTA legislation on consent exemptions, the samples were used without obtaining explicit consent.

## Conflicts of Interest

The authors declare no conflicts of interest.

## Supporting information


Appendix S1.

Appendix S2.

## Data Availability

The datasets generated and/or analysed in this article are available in the University of Glasgow data repository, https://doi.org/10.5525/gla.researchdata.1308; http://researchdata.gla.ac.uk/784/. The raw sequencing files are available in the ArrayExpress data repository (ArrayExpress accession code: E‐MTAB‐11955).
