# Supplementary material for: Elucidation of Dysregulated Pathways Associated With Hypoxia in Oestrogen Receptor‐Negative Breast Cancer
Source: Cancer Med. 2024 Dec 11;13(23):e70274. doi: 10.1002/cam4.70274 (PMC11632397; doi:10.1002/cam4.70274)
Supplement: Supplementary file 1 — Appendix S1. [file CAM4-13-e70274-s002.docx]

**Supplementary Figures**


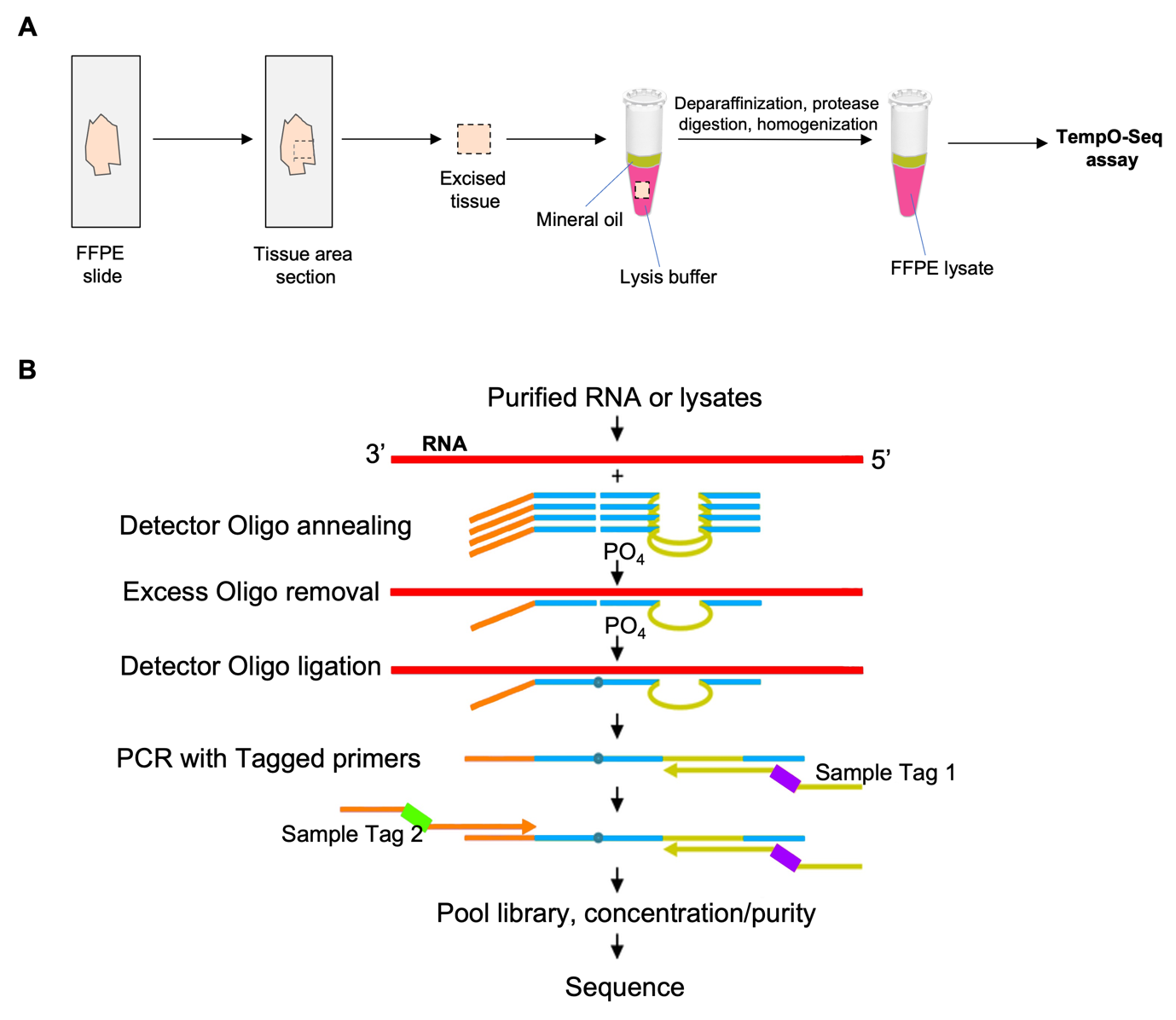


Figure S1. Processing FFPE samples for the TempO-Seq assay

**[A]** An interest area is manually scraped from mounted FFPE sections. The tissue is added directly into FFPE lysis buffer, overlaid with mineral oil, and then heated at 95°C for 5 minutes. FFPE Protease is added, and the sample is incubated for 30 minutes and manually homogenized. The processed lysate is then ready for input directly into the annealing step of the TempO-Seq assay. **[B]** Schematic of the TempO-Seq detector oligo annealing and ligation process. Adapted from Yeakley et al. (34).


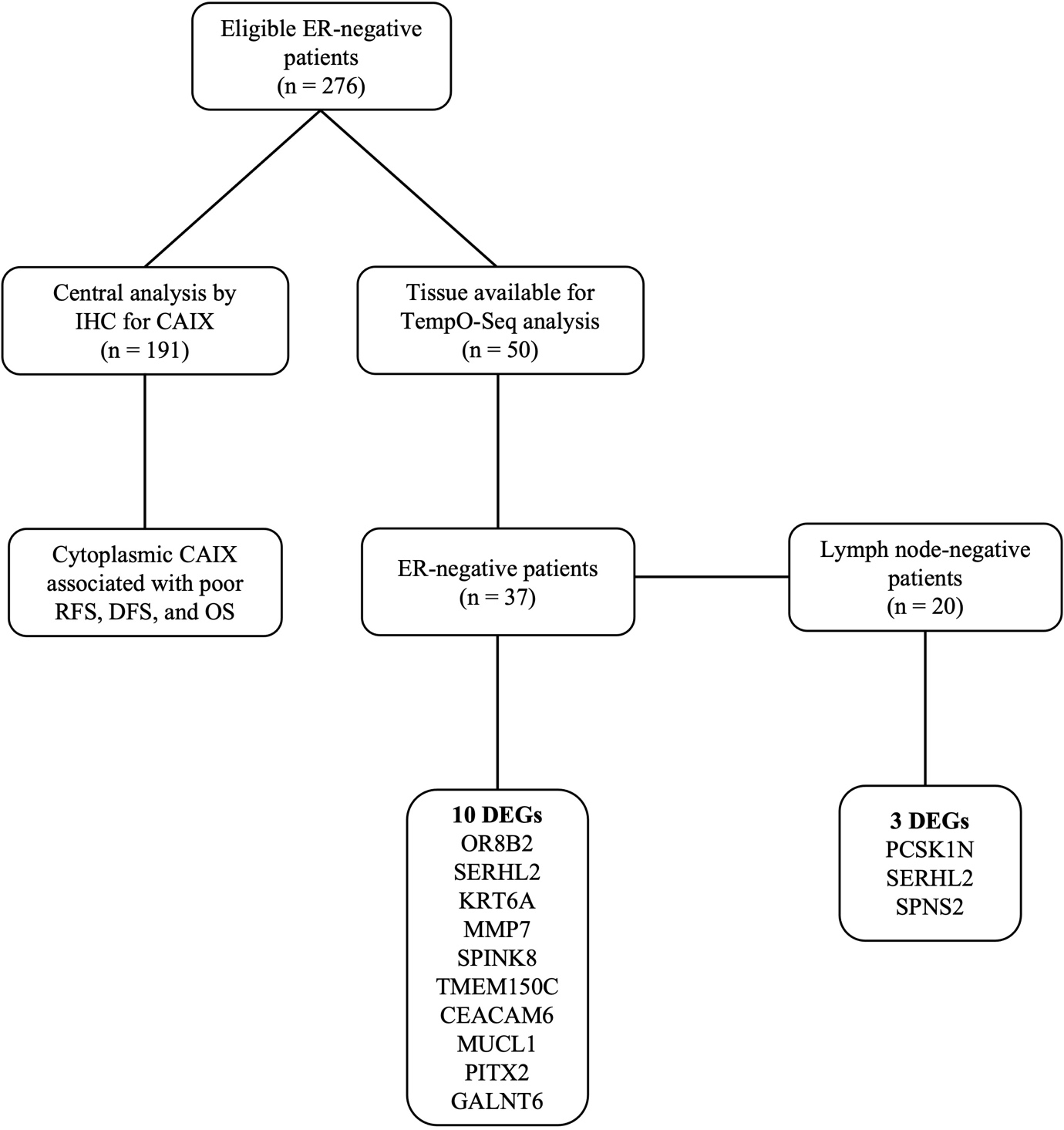


**Figure S2. A diagram of proposed model based on the findings**


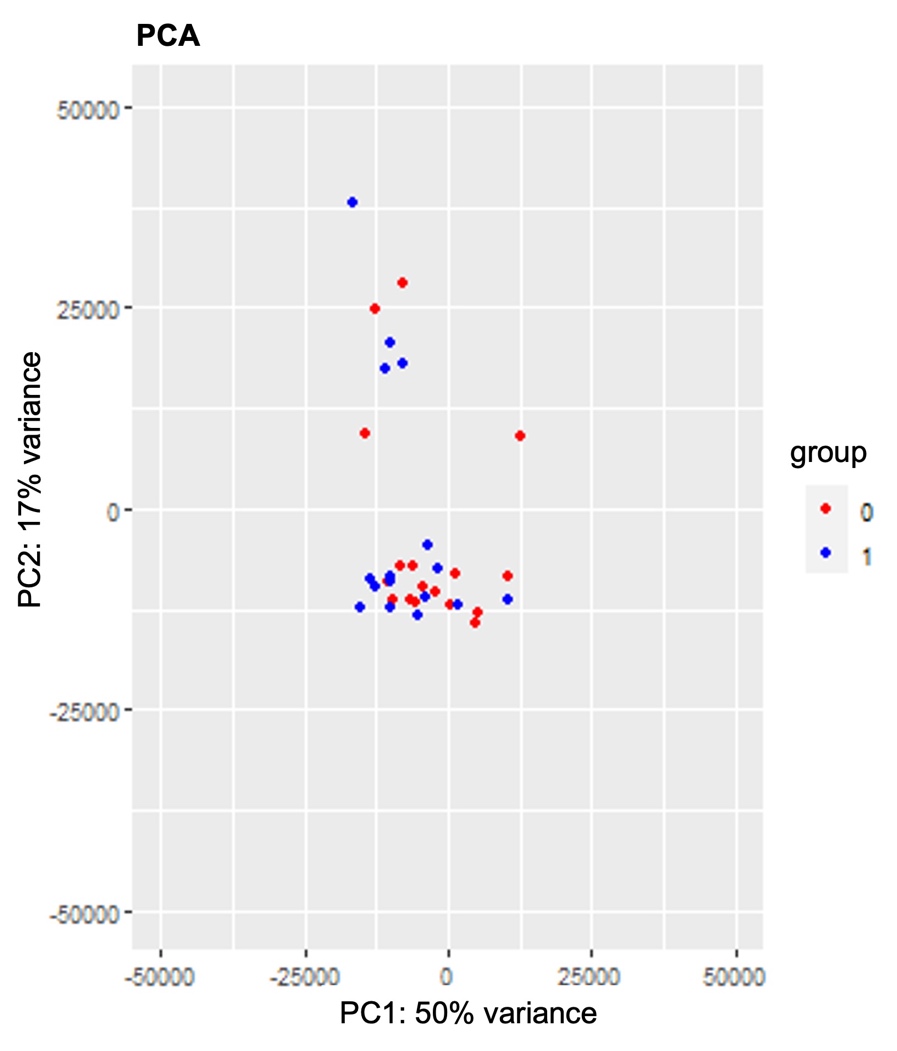


Figure S3. Principal component analysis scatter plots for ER-negative tumour

PCA plots of differential expression genes identified between tumour with high (blue) and low (red) cytoplasmic CAIX expression. Two outliers were excluded from the plot. Gene counts were obtained from full transcriptome sequencing performed by TempO-Seq in a subset of the ER-negative cohort.

**
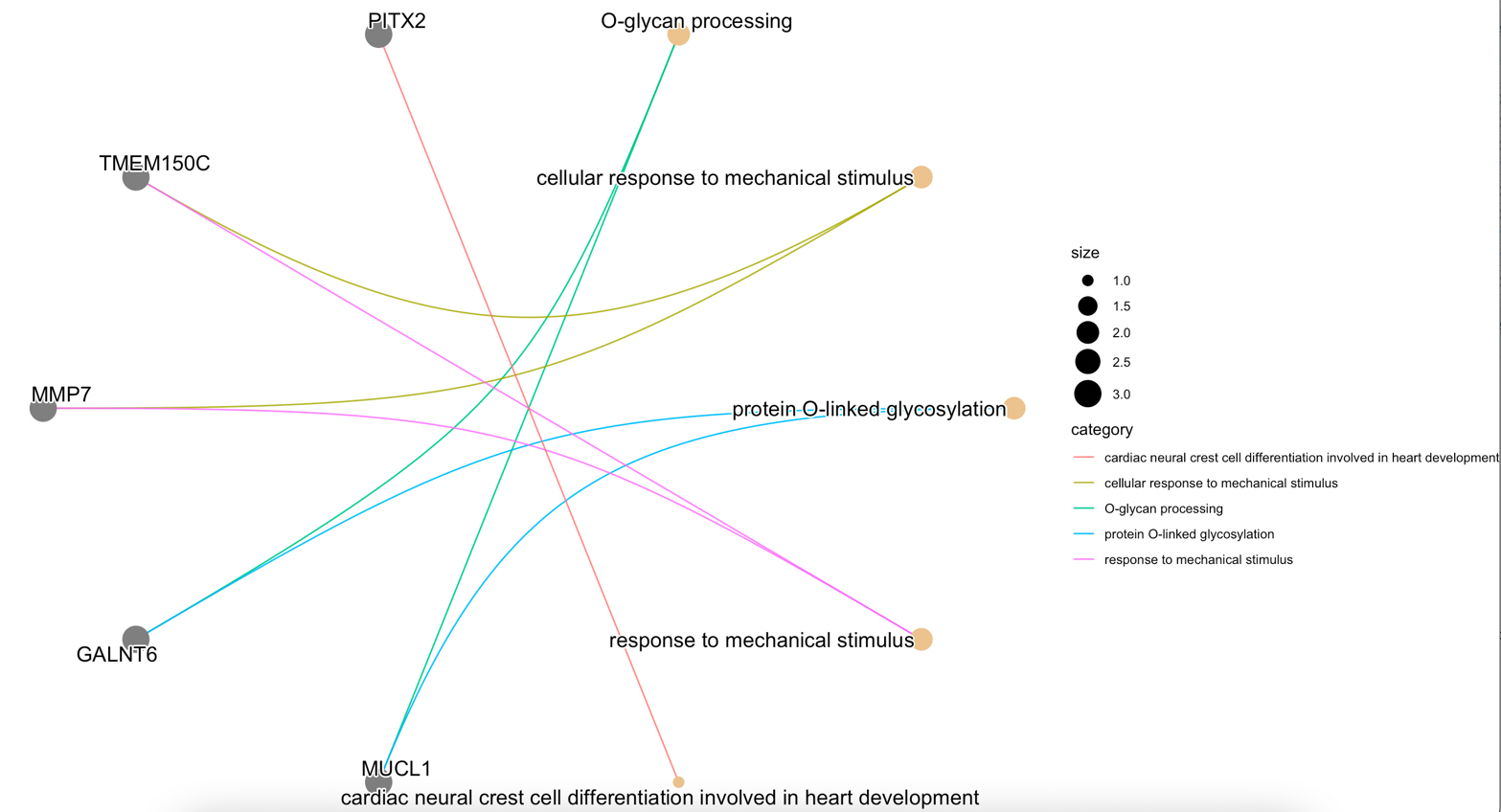
**

**Figure S4. Enrichment cnetplot for differential expression genes**

Cnetplot depicts **relationships of** enriched genes with the corresponding enriched pathways. Relationships as a network diagram with associated data to colour nodes.


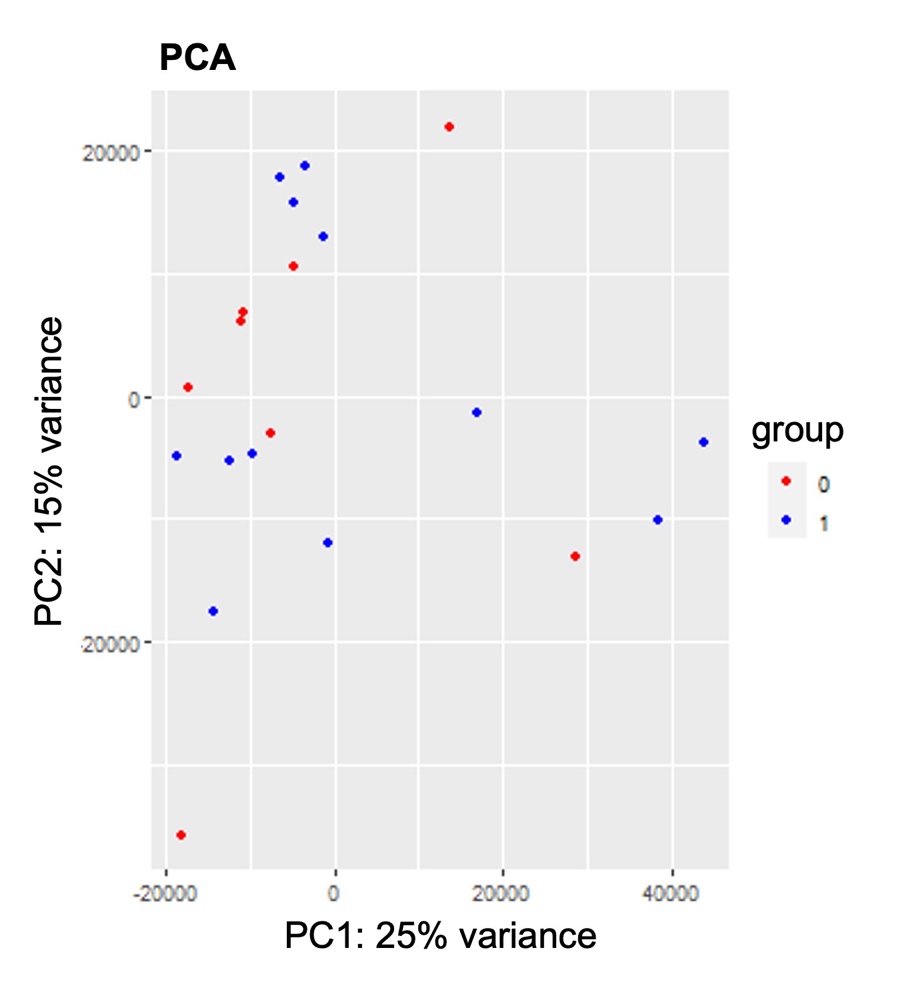


Figure S5. Principal component analysis scatter plots in node-negative group

PCA plots of differential expression genes identified between tumour with high (blue) and low (red) cytoplasmic CAIX expression. Two outliers were excluded from the plot.
