# Supplementary material for: Elucidation of Dysregulated Pathways Associated With Hypoxia in Oestrogen Receptor‐Negative Breast Cancer
Source: Cancer Med. 2024 Dec 11;13(23):e70274. doi: 10.1002/cam4.70274 (PMC11632397; doi:10.1002/cam4.70274)
Supplement: Supplementary file 2 — Appendix S2. [file CAM4-13-e70274-s001.docx]

**Supplementary Tables**

**Table S1. A quantification of CAIX IHC data (n = 191)**

| TMA ID | TMA code | Cytoplasmic CAIX | Membranous CAIX |
| --- | --- | --- | --- |
| 34 | A | 2.5 | 0 |
| 49 | A | 0 | 0 |
| 59 | A | 0 | 0 |
| 61 | A | 0 | 0 |
| 67 | A | 0 | 0 |
| 102 | A | 0 | 0 |
| 159 | A | 0 | 52.5 |
| 215 | A | 47.5 | 67.5 |
| 217 | A | 0 | 0 |
| 220 | A | 0 | 0 |
| 226 | B | 22.5 | 10 |
| 227 | B | 3.33 | 5 |
| 230 | B | 12.5 | 20 |
| 231 | B | 0 | 0 |
| 232 | B | 72.5 | 40 |
| 233 | B | 47.5 | 60 |
| 234 | B | 0 | 0 |
| 235 | B | 0 | 0 |
| 236 | B | 35 | 25 |
| 238 | B | 0 | 0 |
| 239 | B | 217.5 | 260 |
| 242 | B | 0 | 0 |
| 243 | B | 0 | 0 |
| 245 | B | 75 | 120 |
| 248 | B | 30 | 32.5 |
| 251 | B | 42.5 | 62.5 |
| 254 | B | 0 | 0 |
| 255 | B | 10 | 0 |
| 257 | B | 80 | 108.33 |
| 260 | B | 2.5 | 0 |
| 261 | B | 185 | 210 |
| 264 | B | 66.67 | 101.67 |
| 265 | B | 0 | 0 |
| 266 | B | 95 | 40 |
| 274 | B | 145 | 125 |
| 523 | B | 30 | 48.33 |
| 524 | B | 0 | 0 |
| 526 | B | 0 | 0 |
| 529 | B | 0 | 0 |
| 530 | B | 33.33 | 55 |
| 534 | B | 123.33 | 171.67 |
| 535 | B | 97.5 | 137.5 |
| 540 | B | 0 | 0 |
| 545 | B | 0 | 0 |
| 1594 | B | 0 | 0 |
| 1596 | B | 126.67 | 108.33 |
| 1598 | B | 10 | 10 |
| 1600 | B | 16.67 | 33.33 |
| 1601 | B | 5 | 1.67 |
| 1602 | B | 0 | 0 |
| 1603 | B | 11.67 | 5 |
| 1604 | B | 0 | 0 |
| 1605 | B | 17.5 | 22.5 |
| 1607 | B | 152.5 | 185 |
| 1608 | B | 0 | 0 |
| 1611 | B | 5 | 6.67 |
| 1612 | B | 125 | 163.33 |
| 1613 | B | 0 | 0 |
| 1614 | B | 86.67 | 61.67 |
| 1615 | B | 0 | 0 |
| 1616 | B | 80 | 45 |
| 1619 | B | 0 | 0 |
| 1620 | B | 0 | 0 |
| 1632 | B | 6.67 | 10 |
| 1634 | B | 0 | 0 |
| 1635 | B | 0 | 0 |
| 1636 | B | 8.33 | 11.67 |
| 1638 | B | 0 | 0 |
| 1639 | B | 13.33 | 16.67 |
| 1640 | B | 45 | 70 |
| 1641 | B | 15 | 0 |
| 1643 | B | 27.5 | 17.5 |
| 1644 | B | 0 | 0 |
| 1648 | B | 0 | 0 |
| 1650 | B | 1.67 | 1.67 |
| 1651 | B | 17.5 | 22.5 |
| 1653 | B | 2.5 | 2.5 |
| 1655 | B | 85 | 90 |
| 1662 | B | 90 | 90 |
| 1664 | B | 0 | 0 |
| 1668 | B | 83.33 | 38.33 |
| 1673 | B | 0 | 0 |
| 1675 | B | 10 | 17.5 |
| 1676 | B | 0 | 0 |
| 1682 | B | 0 | 0 |
| 1687 | B | 0 | 0 |
| 1690 | B | 0 | 0 |
| 1692 | B | 111.67 | 136.67 |
| 1693 | B | 0 | 0 |
| 1697 | B | 0 | 0 |
| 1698 | B | 130 | 170 |
| 1700 | B | 10 | 11.67 |
| 1703 | B | 7.5 | 7.5 |
| 1705 | B | 0 | 0 |
| 1706 | B | 0 | 0 |
| 1708 | B | 46.67 | 38.33 |
| 1709 | B | 0 | 0 |
| 1710 | B | 16.67 | 16.67 |
| 1711 | B | 0 | 0 |
| 1712 | B | 0 | 0 |
| 1713 | B | 31.67 | 35 |
| 1715 | B | 2.5 | 5 |
| 1718 | B | 0 | 0 |
| 1719 | B | 122.5 | 235 |
| 1723 | B | 145 | 190 |
| 1724 | B | 0 | 0 |
| 1725 | B | 10 | 10 |
| 1728 | B | 0 | 0 |
| 1729 | B | 0 | 0 |
| 1731 | B | 90 | 35 |
| 348 | C | 2.78 | 1.67 |
| 1126 | D | 0 | 0 |
| 1201 | D | 0 | 0 |
| 1218 | D | 1.67 | 3.33 |
| 1390 | D | 35 | 30 |
| 1408 | D | 0 | 0 |
| 1425 | D | 0 | 0 |
| 1561 | D | 0 | 0 |
| 370 | F | 47.5 | 72.5 |
| 371 | F | 0 | 0 |
| 372 | F | 46.67 | 78.33 |
| 373 | F | 38.33 | 45 |
| 377 | F | 0 | 0 |
| 381 | F | 26.67 | 43.33 |
| 382 | F | 0 | 0 |
| 383 | F | 0 | 0 |
| 384 | F | 68.33 | 100 |
| 387 | F | 0 | 0 |
| 390 | F | 0 | 0 |
| 392 | F | 35 | 42.5 |
| 393 | F | 0 | 0 |
| 405 | F | 43.33 | 60 |
| 407 | F | 25 | 41.67 |
| 411 | F | 66.67 | 100 |
| 412 | F | 0 | 0 |
| 415 | F | 0 | 0 |
| 896 | F | 115 | 167.5 |
| 898 | F | 16.67 | 30 |
| 899 | F | 51.67 | 88.33 |
| 906 | F | 27.5 | 35 |
| 912 | F | 70 | 92.5 |
| 913 | F | 0 | 0 |
| 917 | F | 0 | 0 |
| 918 | F | 3.33 | 8.33 |
| 919 | F | 0 | 0 |
| 920 | F | 3.33 | 6.67 |
| 921 | F | 23.33 | 43.33 |
| 924 | F | 0 | 0 |
| 925 | F | 1.67 | 5 |
| 926 | F | 0 | 0 |
| 928 | F | 0 | 0 |
| 931 | F | 11.67 | 33.33 |
| 932 | F | 11.67 | 18.33 |
| 933 | F | 0 | 0 |
| 934 | F | 0 | 0 |
| 940 | F | 1.67 | 5 |
| 941 | F | 3.33 | 3.33 |
| 942 | F | 0 | 0 |
| 947 | F | 85 | 145 |
| 949 | F | 5 | 8.33 |
| 950 | F | 28.33 | 43.33 |
| 953 | F | 3.33 | 6.67 |
| 957 | F | 38.33 | 75 |
| 960 | F | 137.5 | 155 |
| 961 | F | 1.67 | 5 |
| 962 | F | 1.67 | 1.67 |
| 964 | F | 6.67 | 15 |
| 966 | F | 15 | 26.67 |
| 976 | F | 16.67 | 26.67 |
| 978 | F | 1.67 | 1.67 |
| 980 | F | 0 | 0 |
| 981 | F | 3.33 | 5 |
| 983 | F | 6.67 | 13.33 |
| 986 | F | 1.67 | 5 |
| 987 | F | 15 | 23.33 |
| 988 | F | 28.33 | 55 |
| 994 | F | 10 | 20 |
| 995 | F | 0 | 0 |
| 999 | F | 15 | 30 |
| 1002 | F | 25 | 35 |
| 1003 | F | 0 | 0 |
| 1005 | F | 5 | 10 |
| 1009 | F | 13.33 | 26.67 |
| 1010 | F | 75 | 153.33 |
| 1011 | F | 1.67 | 3.33 |
| 1016 | F | 25 | 41.67 |
| 1017 | F | 66.67 | 101.67 |
| 1019 | F | 23.33 | 45 |
| 1021 | F | 20 | 36.67 |
| 1022 | F | 16.67 | 28.33 |
| 1025 | F | 0 | 0 |

**Table S2. Primer sequences, product size and location for target genes**

| Target gene | Gene ID | Forward primer | Reverse primer | Product size | Tm | Location |
| --- | --- | --- | --- | --- | --- | --- |
| SERHL2 | 253190 | GGCTCTCGTCCCATTACAGC | CTGCCACAACTCTTCGGATCT | 78 | 61.8/62.4 | 209-286 |
| CEACAM6 | 4680 | TCAATGGGACGTTCCAGCAAT | CACTCCAATCGTGATGCCGA | 194 | 62.0/62.3 | 818-1011 |
| MUCL1 | 118430 | TCTGCCCAGAATCCGACAAC | AATGTCTTTACGAGCAGTGGTAG | 168 | 61.9/60 | 52-219 |
| PITX2 | 5308 | TGTGGACCAACCTTACGGAAG | ATGAGCCCATTGAACTGCGG | 133 | 61.7/62.9 | 221-353 |
| GALNT6 | 11226 | ACAGCGTCCTACACACCAC | CTTCTCCTTTAGGTGCTCCTCT | 89 | 61.6/60.6 | 593-681 |
| MMP7 | 4316 | GAGTGAGCTACAGTGGGAACA | CTATGACGCGGGAGTTTAACAT | 158 | 61.1/60.2 | 81-238 |
| SPNS2 | 124976 | ATGGCTCCGAGATATGAAGGC | CTGTCTTCTGCACAACTTGGG | 146 | 61.5/61.1 | 903-1048 |

Table S3. The clinicopathological characteristics of ER-negative cohort (n=191)

| Clinicopathological characteristics | Patients, n (%) |
| --- | --- |
| Age (≤50/ >50 years) | 72(38)/119(62) |
| Size (≤ 20/ 21–50/ >50 mm) | 97(51)/86(45)/8(4) |
| Grade (I/ II/ III) | 5(2)/36(19)/150(79) |
| Lymph node status (negative/ Positive) | 98(51)/93(49) |
| PR status (negative/ Positive) | 184(96)/7(4) |
| Her-2 status (negative/ Positive) | 129(69)/59(31) |
| Ki67 (proliferative index) (low/ High) | 117(66)/60(34) |
| Molecular subtype (TNBC/ Her-2) | 124(66)/57(30) |
| Adjuvant chemotherapy (no/ Yes) | 63(33)/127(67) |
| Adjuvant radiotherapy (No/ Yes) | 84(44)/106(56) |
| No recurrence/ Local/ Distant/ Both | 126(68)/15(8)/43(24)/2(1) |
| Alive/ Cancer death/ Non-cancer death | 97(52)/58(31)/29(16)/3(1) |

| Clinicopathological  characteristics | Cytoplasmic CAIX | | |
| --- | --- | --- | --- |
|  | **Low expression**  **(n=130) (68%)** | **High expression**  **(n=61) (32%)** | **P-value** |
| Age (≤50 />50 years) | 53(41)/77(59) | 19(31)/42(69) | 0.197 |
| Tumour size (mm) (≤ 20/ 21-50/ >50) | 70(54)/54(42)/6(4) | 27(44)/32(53)/2(3) | 0.358 |
| Grade (I / II / III) | 4(3)/27(21)/99(76) | 1(1)/9(15)/51(84) | 0.239 |
| Nodal status (Negative / Positive) | 64(49)/66(51) | 34(56)/27(44) | 0.401 |
| PR status (Negative / Positive) | 125(96)/5(4) | 59(97)/2(3) | 0.844 |
| Her-2 status (Negative / Positive) | 89(70)/39(30) | 40(67)/20(33) | 0.694 |
| Ki67 index (Low / High) | 86(71)/36(29) | 31(56)/24(44) | 0.069 |
| Lymphatic vessel invasion (No/ Yes) | 52(64)/29(36) | 22(61)/14(39) | 0.750 |
| Blood vessel invasion (No/ Yes) | 70(86)/11(14) | 29(81)/7(19) | 0.425 |
| Tumour necrosis (Low/ High) | 36(28)/94(72) | 6(10)/55(90) | 0.003* |
| Klintrup-Mäkinen grade (Low/ High) | 4(3)/56(43)/51(39)/19(15) | 0(0)/23(38)/34(56)/4(6) | 0.754 |
| CD68+ (Low/ Moderate/ High) | 22(31)/20(27)/30(42) | 14(44)/6(18)/12(38) | 0.347 |
| CD8+ (Low/ Moderate/ High) | 18(25)/16(22)/38(53) | 8(24)/7(21)/18(55) | 0.886 |
| CD138+ (Low/ Moderate/ High) | 33(46)/6(8)/33(46) | 13(41)/3(9)/16(50) | 0.646 |
| Tumour stroma percentage (Low/ High) | 87(67)/43(33) | 39(64)/22(36) | 0.685 |
| Tumour budding (Low/ High) | 101(78)/29(22) | 48(79)/13(21) | 0.877 |
| Adjuvant chemotherapy (No/ Yes) | 37(29)/92(71) | 26(43)/35(57) | 0.059 |
| Adjuvant radiotherapy (No/ Yes) | 53(41)/76(59) | 31(51)/30(49) | 0.208 |

Table S4. Relationship between cytoplasmic CAIX protein expression and clinicopathological parameters in ER-negative cohort (n=191)

**Table S5. Clinicopathological characteristics summary of ER-negative cohort utilized in transcriptomic analysis (n=37)**

| Clinicopathological characteristics | Patients, n (%) |
| --- | --- |
| Age (≤50 / >50 years) | 14(38)/23(62) |
| Tumour size (mm) (20/ 21-50/ >50) | 12(32)/22(60)/3(8) |
| Grade (I / II / III) | 2(5)/7(19)28(76) |
| Lymph node status (Negative/ Positive) | 20(54)/17(46) |
| PR status (Negative/ Positive) | 36(97)1(3) |
| Her-2 status (Negative/ Positive) | 27(73)/10(27) |
| Ki67 (Proliferative index) (Low/ High) | 26(72)/10(28) |
| Adjuvant chemotherapy (No/ Yes) | 12(32)/25(68) |
| Adjuvant radiotherapy (No/ Yes) | 19(51)/18(49) |

Table S6. Clinicopathological characteristics of the high compared to the low cytoplasmic CAIX protein expression in ER-negative cohort utilized in transcriptomic analysis (n=37)

| Clinicopathological  characteristics | Cytoplasmic CAIX | | |
| --- | --- | --- | --- |
|  | **Low (n=21)** | **High (n=16)** | **P*-*value** |
| Age (< 50 / > 50) | 9(43)/12(57) | 5(31)/11(69) | 0.469 |
| Tumour size (mm) (≤ 20/ 21-50/ >50) | 6(29)/13(62)/2(9) | 6(38)/9(56)/1(6) | 0.538 |
| Grade (I / II / III) | 1(5)/5(24)/15(71) | 1(6)/2(13)/13(81) | 0.660 |
| Lymph node status (Negative/ Positive) | 8(38)/13(62) | 12(75)/4(25) | 0.023* |
| PR (Negative/ Positive) | 21(100)/0(0) | 15(94)/1(6) | 0.191 |
| Her-2 (Negative/ Positive) | 16(76)/5(24) | 11(69)/5(31) | 0.615 |

**Table S7. Pathways enrichment analysis in ER-negative cohort**

| Pathway enrichment | Matching proteins | FDR |
| --- | --- | --- |
| WNT signalling pathway (KEGG) | MMP7 | 0.0332 |
| Extracellular matrix organization (Reactome) | MMP7, CEACAM6 | 6.51e-05 |
| Degradation of extracellular matrix (Reactome) | MMP7 | 4.78e-05 |
| Disease of glycosylation (Reactome) | MUCL1 | 0.0476 |

| Pathway enrichment | Matching proteins | FDR |
| --- | --- | --- |
| Sphingolipid de novo biosynthesis (Reactome) | SPNS2 | 0.0277 |
| Insulin processing (Reactome) | PCSK1N | 0.0160 |

**Table S8. Pathways enrichment analysis in node-negative group**

**Table S9. Gene expression in hypoxic and normoxic MDA-MB-231 cells**

|  | C | 4N | 4H | 8N | 8H | 16N | 16H | 24N | 24H | 48N | 48H |
| --- | --- | --- | --- | --- | --- | --- | --- | --- | --- | --- | --- |
| CEACAM | 34.98864 | Undetermined | Undetermined | 33.97851 | 35.8588 | Undetermined | 34.67249 | 34.57594 | 32.99398 | Undetermined | Undetermined |
| normalise to GAPDH | 34.85369 | 35.36623 | 33.64743 | 37.65061 | 37.84676 | Undetermined | 34.62585 | 28.52756 | 34.06573 | 33.09654 | 35.36257 |
| AVE | 34.92117 | 35.36623 | 33.64743 | 35.81456 | 36.85278 | #DIV/0! | 34.64917 | 31.55175 | 33.52986 | 33.09654 | 35.36257 |
| power | 1.44E-06 | 1.22E-06 | 2.03E-05 | 7.58E-07 | 7.8E-07 | #DIV/0! | 3.78E-07 | 9.5E-06 | 3.11E-06 | 7.58E-06 | 2.24E-06 |
| ratio to control | 1.000 | 0.849005 | 14.04981 | 0.525236 | 0.540381 | #DIV/0! | 0.262289 | 6.587667 | 2.153634 | 5.256745 | 1.555478 |
| raio of H/N | |  | 16.54856 |  | 1.028835 |  | #DIV/0! |  | 0.326919 |  | 0.295901 |
|  |  |  |  |  |  |  |  |  |  |  |  |
|  | C | 4N | 4H | 8N | 8H | 16N | 16H | 24N | 24H | 48N | 48H |
| MCUL1 | 36.44931 | 38.21013 | 36.2667 | 31.92664 | 32.52111 | 34.0145 | 33.67369 | 31.93475 | 33.04012 | 33.1187 | Undetermined |
| normalise to GAPDH | 33.39519 | 32.81474 | 35.46793 | 33.40972 | 31.72034 | Undetermined | 32.93802 | 34.23114 | 33.36592 | 32.34661 | 36.77103 |
| AVE | 34.92225 | 35.51243 | 35.86731 | 32.66818 | 32.12073 | 34.0145 | 33.30586 | 33.08294 | 33.20302 | 32.73265 | 36.77103 |
| power | 1.44E-06 | 1.11E-06 | 4.35E-06 | 6.71E-06 | 2.07E-05 | 6.23E-06 | 9.6E-07 | 3.29E-06 | 3.9E-06 | 9.76E-06 | 8.45E-07 |
| ratio to control | 1.000 | 0.768 | 3.018193 | 4.654101 | 14.37203 | 4.318642 | 0.666016 | 2.280991 | 2.703248 | 6.76993 | 0.58641 |
| raio of H/N | |  | 3.931181 |  | 3.088036 |  | 0.154219 |  | 1.18512 |  | 0.08662 |
|  |  |  |  |  |  |  |  |  |  |  |  |
|  | C | 4N | 4H | 8N | 8H | 16N | 16H | 24N | 24H | 48N | 48H |
| GALNT6 | 22.14155 | 22.33428 | 24.81578 | 22.43697 | 23.5425 | 24.29192 | 21.45739 | 21.98953 | 22.91705 | 23.27982 | 24.24912 |
| normalise to GAPDH | 22.28487 | 22.29398 | 24.57422 | 22.23178 | 23.65408 | 23.94333 | 21.49368 | 22.22913 | 22.96353 | 23.25258 | 24.05894 |
| AVE | 22.21321 | 22.31413 | 24.695 | 22.33437 | 23.59829 | 24.11763 | 21.47553 | 22.10933 | 22.94029 | 23.2662 | 24.15403 |
| power | 0.009654 | 0.010404 | 0.010042 | 0.00866 | 0.007619 | 0.005936 | 0.003497 | 0.006613 | 0.004788 | 0.006905 | 0.005311 |
| ratio to control | 1.000 | 1.078 | 1.040 | 0.897051 | 0.789265 | 0.614892 | 0.362212 | 0.685024 | 0.49599 | 0.715265 | 0.550168 |
| raio of H/N | |  | 0.96525 |  | 0.879845 |  | 0.589066 |  | 0.724047 |  | 0.769181 |
|  |  |  |  |  |  |  |  |  |  |  |  |
|  | C | 4N | 4H | 8N | 8H | 16N | 16H | 24N | 24H | 48N | 48H |
| SERHL2 | 25.32706 | 25.71145 | 27.37149 | 25.48151 | 26.39811 | 26.2762 | 23.91724 | 25.30749 | 25.53728 | 26.00251 | 26.45164 |
| normalise to GAP | 25.2426 | 25.62494 | 27.70126 | 25.45591 | 26.2282 | 26.29361 | 23.89099 | 25.3942 | 25.68835 | 25.70361 | 26.4482 |
| AVE | 25.28483 | 25.66819 | 27.53638 | 25.46871 | 26.31315 | 26.2849 | 23.90412 | 25.35085 | 25.61281 | 25.85306 | 26.44992 |
| power | 0.001148 | 0.001017 | 0.001401 | 0.000986 | 0.001161 | 0.001322 | 0.00065 | 0.000699 | 0.000751 | 0.001149 | 0.001082 |
| ratio to control | 1.000 | 0.886 | 1.220269 | 0.858891 | 1.010692 | 1.150892 | 0.565637 | 0.608926 | 0.654055 | 1.000911 | 0.941917 |
| raio of H/N | |  | 1.377123 |  | 1.17674 |  | 0.491477 |  | 1.074113 |  | 0.94106 |
|  |  |  |  |  |  |  |  |  |  |  |  |
|  | C | 4N | 4H | 8N | 8H | 16N | 16H | 24N | 24H | 48N | 48H |
| MMP7 | 29.14946 | 28.83572 | 31.11649 | 29.52767 | 30.29714 | 29.57628 | 27.00558 | 29.27079 | 29.50417 | 30.55018 | 30.76883 |
| normalise to GAP | 28.20449 | 29.34355 | 30.70562 | 28.73924 | 29.06583 | 30.12809 | 27.11237 | 28.92021 | 29.19259 | 29.84162 | 31.90551 |
| AVE | 28.67698 | 29.08963 | 30.91105 | 29.13345 | 29.68148 | 29.85218 | 27.05898 | 29.0955 | 29.34838 | 30.1959 | 31.33717 |
| power | 0.000109 | 9.5E-05 | 0.000135 | 7.78E-05 | 0.000112 | 0.000111 | 7.29E-05 | 5.22E-05 | 5.64E-05 | 5.66E-05 | 3.65E-05 |
| ratio to control | 1.000 | 0.868 | 1.235136 | 0.711015 | 1.027516 | 1.019328 | 0.666757 | 0.476923 | 0.515507 | 0.517855 | 0.334151 |
| raio of H/N | |  | 1.422496 |  | 1.445139 |  | 0.654114 |  | 1.080901 |  | 0.645259 |
|  |  |  |  |  |  |  |  |  |  |  |  |
|  | C | 4N | 4H | 8N | 8H | 16N | 16H | 24N | 24H | 48N | 48H |
| PITX2 | 27.46153 | 27.00895 | 29.58884 | 26.74414 | 27.73978 | 27.45745 | 25.80675 | 26.53522 | 27.37325 | 26.93703 | 28.38633 |
| normalise to GAP | 26.93379 | 26.84123 | 29.5011 | 26.56591 | 27.87202 | 26.96316 | 25.48643 | 26.39748 | 27.63269 | 26.74466 | 28.98173 |
| AVE | 27.19766 | 26.92509 | 29.54497 | 26.65502 | 27.8059 | 27.2103 | 25.64659 | 26.46635 | 27.50297 | 26.84084 | 28.68403 |
| power | 0.000305 | 0.000426 | 0.000348 | 0.000433 | 0.000412 | 0.000696 | 0.000194 | 0.000323 | 0.000203 | 0.00058 | 0.00023 |
| ratio to control | 1.000 | 1.396 | 1.141897 | 1.421157 | 1.352308 | 2.281813 | 0.636532 | 1.058239 | 0.664413 | 1.900474 | 0.753872 |
| raio of H/N | |  | 0.817879 |  | 0.951554 |  | 0.278959 |  | 0.627848 |  | 0.396676 |
|  |  |  |  |  |  |  |  |  |  |  |  |
|  | C | 4N | 4H | 8N | 8H | 16N | 16H | 24N | 24H | 48N | 48H |
| SNPS2 | 24.82244 | 25.3672 | 26.98258 | 25.56833 | 26.82364 | 27.47959 | 26.77459 | 25.9276 | 25.802 | 27.15367 | 27.9234 |
| normalise to GAP | 24.81195 | 25.14831 | 27.16917 | 25.20571 | 26.641 | 27.75386 | 26.91493 | 25.88701 | 25.70719 | 27.2678 | 27.4467 |
| AVE | 24.81719 | 25.25775 | 27.07588 | 25.38702 | 26.73232 | 27.61672 | 26.84476 | 25.90731 | 25.75459 | 27.21074 | 27.68505 |
| power | 0.001588 | 0.001352 | 0.001928 | 0.001044 | 0.000868 | 0.000525 | 8.46E-05 | 0.000475 | 0.000681 | 0.000448 | 0.000459 |
| ratio to control | 1 | 0.851656 | 1.214245 | 0.657289 | 0.546592 | 0.330631 | 0.053277 | 0.299419 | 0.428707 | 0.282436 | 0.289354 |
| raio of H/N | |  | 1.425746 |  | 0.831586 |  | 0.161138 |  | 1.431794 |  | 1.024494 |
